# Efficient molasses fermentation under high salinity by inocula of marine and terrestrial origin

**DOI:** 10.1186/s13068-017-0701-8

**Published:** 2017-01-31

**Authors:** Alberto Scoma, Marta Coma, Frederiek-Maarten Kerckhof, Nico Boon, Korneel Rabaey

**Affiliations:** 1 Center of Microbial Ecology and Technology (CMET), University of Gent, Coupure Links 653, 9000 Ghent, Belgium; 2 Centre for Sustainable Chemical Technologies (CSCT), University of Bath, Claverton Down, Bath, BA2 7AY UK; 3 Center for Geomicrobiology, Aarhus University, Ny Munkegade 116, 8000 Aarhus C, Denmark

**Keywords:** Fermentation, Biorefineries, Bulk chemicals, Brines, Halophiles, Hydrogen, Methane, VFA, Carboxylate, Anaerobic digestion

## Abstract

**Background:**

Molasses is a dense and saline by-product of the sugar agroindustry. Its high organic content potentially fuels a myriad of renewable products of industrial interest. However, the biotechnological exploitation of molasses is mainly hampered by the high concentration of salts, an issue that is nowadays tackled through dilution. In the present study, the performance of microbial communities derived from marine sediment was compared to that of communities from a terrestrial environment (anaerobic digester sludge). The aim was to test whether adaptation to salinity represented an advantage for fermenting molasses into renewable chemicals such as volatile fatty acids (VFAs) although high sugar concentrations are uncommon to marine sediment, contrary to anaerobic digesters.

**Results:**

Terrestrial and marine microbial communities were enriched in consecutive batches at different initial pH values (pH_i_; either 6 or 7) and molasses dilutions (equivalent to organic loading rates (OLRs) of 1 or 5 g_COD_ L^−1^ d^−1^) to determine the best VFA production conditions. Marine communities were supplied with NaCl to maintain their native salinity. Due to molasses inherent salinity, terrestrial communities experienced conditions comparable to brackish or saline waters (20–47 mS cm^−1^), while marine conditions resembled brine waters (>47 mS cm^−1^). Enrichments at optimal conditions of OLR 5 g_COD_ L^-1^ d^-1^ and pH_i_ 7 were transferred into packed-bed biofilm reactors operated continuously. The reactors were first operated at 5 g_COD_ L^-1^ d^-1^, which was later increased to OLR 10 g_COD_ L^−1^ d^−1^. Terrestrial and marine reactors had different gas production and community structures but identical, remarkably high VFA bioconversion yields (above 85%) which were obtained with conductivities up to 90 mS cm^−1^. COD-to-VFA conversion rates were comparable to the highest reported in literature while processing other organic leftovers at much lower salinities.

**Conclusions:**

Although salinity represents a major driver for microbial community structure, proper acclimation yielded highly efficient systems treating molasses, irrespective of the inoculum origin. Selection of equivalent pathways in communities derived from different environments suggests that culture conditions select for specific functionalities rather than microbial representatives. Mass balances, microbial community composition, and biochemical analysis indicate that biomass turnover rather than methanogenesis represents the main limitation to further increasing VFA production with molasses. This information is relevant to moving towards molasses fermentation to industrial application.

**Electronic supplementary material:**

The online version of this article (doi:10.1186/s13068-017-0701-8) contains supplementary material, which is available to authorized users.

## Background

Side streams from the agroindustry are attractive renewable feedstocks in the frame of biorefineries due to their abundance and generally low value. To be economically competitive, these streams should contain highly degradable substrates to increase the actual conversion yields of biowastes into chemicals of interest. Molasses is a dense, highly refined by-product produced worldwide derived from the downstream processing of sugar industries [1, 2]. Provided that its organic fraction is almost entirely due to sucrose, molasses application as feedstock in biorefineries would potentially fuel a myriad of microbial pathways. Production of bio-based chemicals using molasses has been investigated, most frequently to produce biofuels such as ethanol, hydrogen, and methane [2 and references therein].

Besides the high concentration of organics, molasses-based biotechnologies met with limited success due to molasses salinity, demanding strong dilutions prior to any biological process [3] and leading to saline waste streams. Reduction of ash, particles, and salts through microfiltration [4] and desalination [5], or use of specific supporting materials for biomass growth [6–8] has been adopted to mitigate salinity. Alternatively, improved microbial conversion efficiency was attempted through co-digestion with other substrates [9–11]. Understanding how to overcome the biocatalytic boundaries imposed by salinity would enhance the bioconversion of molasses constituents. Microbial community acclimation and proper reactor design successfully addressed some of the issues related with salinity [12]. High salt concentrations are tolerated by or even mandatory for a wide range of microbes [13]. Moderately halophilic microbes grow at concentrations between 2 and 20% NaCl (i.e., saline to brine waters, Table 1), while extreme halophiles require at least 15% NaCl [14]. Increasing NaCl concentrations results in higher conductivities, a parameter indicative of the salinity of a liquid medium although not proportionally, as it is affected by the total amount of all dissolved salts and mobile charged ions (including minerals, metals, and unprotonated acids). As cell membranes are permeable to water, very high extracellular salinities impose a significant stress on cell homeostasis and functionality [15]. This eventually selects specific microbial representatives, whose presence becomes descriptive of such niches in the environment (Table 1).

**Table 1:** Salinities and respective conductivities in different ecosystems and microbial domains

NaCl		Conductivity (mS cm^−1^)	Water and microbes	Ecosystem
g L^−1^	%			
0.01	0.001	0.01	Fresh water (non-halophilic microbes)	Rivers, soil, lakes
0.5	0.05	0.47		
1	0.1	0.94	Brackish water (moderately and non-halophilic microbes)	
30	3	28	Saline water (moderately microbes)	Seas, oceans, some lakes
40	4	38		
50	5	47		
100	10	94	Brine water (strictly and moderately halophilic microbes)	Salt lakes and seas; brine lenses; cryopegs
350	35	329		

Salinities do not consider temperature and hydrostatic pressure and are solely based on the effect of NaCl concentration

The high sugar content in molasses represents an additional issue for bioprocessing due to the osmotic pressure it exerts. While a tenfold molasses dilution may reduce sugar concentrations to tolerable levels for microbes (Table 2), dissolved salts would still yield conductivities resembling seawater conditions (Tables 1, 2) thus requiring microbial acclimation. Some industrial bioprocess may be less prone than others to attain efficient acclimation to salts as for ethanol production using molasses and *Saccharomyces cerevisiae*, which was already limited by Na^+^ concentrations resembling brackish waters (>0.5 g L^−1^, Table 1; [16]). Other affected biotechnological processes are lignocellulose fermentation [17] and digestion [18], exopolysaccharide (EPS) production, either positively [19] or negatively [20], wastewater nitrification [21] and, most typically, anaerobic digestion [22–25]. In particular, salinity in molasses inhibits anaerobic digestion [26] as the high K^+^ levels exert multiple negative effects on microbial metabolism (particularly on methanogenesis [27]) thereby favoring carboxylate accumulation [28]. One promising alternative could rely on salinity-acclimated inocula (e.g., marine sediment) coupled with robust biotechnological processes such as anaerobic digestion, as shown by the recent positive results on methanogenesis [29, 30]. As only those microbes with the ability to cope with increased salt concentrations within their genomic portfolio can profitably operate under these conditions, halophiles have been considered for peculiar biotechnological applications [31–33].

**Table 2:** Physicochemical features of the molasses employed in this study and of both terrestrial and marine inocula

Parameter	Feedstock	Inocula	
	Raw molasses	Terrestrial	Marine
pH	5.7 ± 0.2	7.7 ± 0.2	7.94 ± 0.0
Conductivity (mS cm^−1^)	29 ± 5	1.6 ± 0.5	19.1 ± 0.0
Density (g cm^−3^)	1.35 ± 0.05	nd	nd
Total COD (g O_2_ L^−1^)	440 ± 97	13 ± 5	4 ± 0.0
Soluble COD (g O_2_ L^−1^)	403 ± 12	7 ± 2	0.12 ± 0.0
Total sugars COD (g O_2_ L^−1^)	367 ± 81	nd	nd
Total VFA (g O_2_ L^−1^)	20 ± 2	0	0
TKN (g N L^−1^)	26.1 ± 0.8	nd	nd
Total solids (TS) (g L^−1^)	765 ± 6	12 ± 1	477 ± 3^a^
Volatile solids (VS) (g L^−1^)	513 ± 4	11 ± 1	26 ± 1^a^

Chemical analyses were conducted in two independent replicates

^a^Units in g Kg^−1^ as marine sediment was a mixture of microorganisms with sand and sea water

In the present study, microbial tolerance towards high salinity in molasses fermentation devoted to volatile fatty acid (VFA) production was evaluated. Bioconversion of molasses into well-known bio-based precursors such as VFAs has been rather neglected in the literature [34] and generally referred to as a secondary pathway. VFAs are the core intermediates of the so-called carboxylate platform [35], whose increasing importance relies on the multiple choices for VFAs conversion into compounds of industrial interest. Chemical processing of VFAs can yield bulk solvents/fuels such as ketones, aldehydes, and esters, with further steps yielding alcohols and alkanes [35], while biomaterials such as bioplastics are biologically produced using VFAs as preferred carbon sources (e.g., the polymers of the hydroxylated fatty acids between C4 and C8 [36]). While still in its infancy, the carboxylate platform has several advantages over the sugar and syngas platforms, with conversion efficiency the main one [37], and patented technologies applied for biomass conversion into carboxylate salts (the MixAlco™ process [38]). In the present study, the performance of an inoculum derived from an anaerobic digester (hereafter referred to as “terrestrial”) was compared with that of marine sediments (hereafter referred to as “marine”). The marine inoculum was derived from an environment that is physically well distinguished from land and, most importantly, was adapted to conductivities one order of magnitude higher than the terrestrial (Table 2). Adaptation was here defined as the set of inheritable traits featuring a microbial community subjected to long-term exposure to a specific environmental context (e.g., salinity for marine microorganisms), as opposed to acclimation, defined as a change of physiological state to counterbalance an environmental shift.

The main aim of the study was (1) to test whether adaptation (marine) or acclimation (terrestrial) to salinity represented an advantage for the proficient bioconversion of molasses into VFAs; and (2) to define optimal operational conditions to attain high molasses conversion yields into VFAs. Both terrestrial and marine communities were first enriched in sequential batch systems and later on tested under continuous mode of operation in a packed-bed biofilm reactor (PBBR). Results were discussed also with respect to the operational changes imposed by feeding a saline bioresidue.

## Methods

### Feedstock and inocula

Molasses were supplied by AVEBE (Veendam, The Netherlands) and kept at 4 °C until use. A general characterization of molasses features is reported in Table 2, while organic and inorganic compositions are depicted in Additional file 1: Fig. S1. DNA extraction from molasses diluted 1:10 and tested by agarose gel electrophoresis was negative, meaning that raw molasses were virtually sterile.

The terrestrial inoculum derived from a 52 L pilot-scale anaerobic digester treating lignocellulosic material (Ghent BioEconomy, CMET, Belgium) at average conditions of 37 °C, pH of 7.8, 15 mS cm^−1^, and 8 g_COD_ L^−1^ d^−1^. The terrestrial inoculum was diluted 1:10 and kept at 4 °C until use. The marine inoculum was derived from a mixture of samples collected at 300 and 1000 m below surface level at the West Iberian Margin, during a cruise by the R/V Belgica from June 2 to June 10, 2014. The sampling area is located at the slope of the Southwest coast of Portugal (latitude comprising between 37°47′ and 37°58′, longitude 09°05′ and 09°28′). Samples employed in this study were collected from the sub-seafloor (between 2 and 15 cm) and kept at 4 °C prior to use. A general characterization of both inocula is reported in Table 2.

### Enrichments in batch experiments

Enrichments of microbial communities for molasses fermentation were conducted in batch, using serum bottles of 120 mL, with a gas:liquid ratio of 40:80 mL. Tests were carried out at 35 °C under constant reciprocal shaking (90 rpm). Each bottle was inoculated with a fixed quantity of inoculum to obtain a concentration of volatile solids (VS) equal to 1 g L^−1^ for 7 days, after which 15 mL was anaerobically transferred into a new serum bottle for another enrichment cycle. In total, 3 consecutive fermentation batches of 7 days each were conducted. Tests were carried out using 2 organic loadings equal to 7 or 35 grams of COD per liter (g_COD_ L^−1^), equivalent to organic loading rates (OLRs) of 1 or 5 g_COD_ L^−1^ d^−1^, with an initial pH (pH_i_) set at either 6 or 7. Variations in pH were smoothed by supplying 10 mL of a 2 M phosphate buffer (24.3% and 6.4% for pH 6 and 11.7 and 30.9% for pH 7 of NaH_2_PO_4_·H_2_O and Na_2_HPO_4_·7H_2_O, respectively), manually correcting the pH to either 6 or 7 when necessary at every sampling time (i.e., day 1, 2, and 4). Dilution of inocula and/or substrates to the working volume was done using tap water in terrestrial cultures, and tap water was supplied with NaCl for marine cultures (22.8 g L^−1^ [39]) in order to preserve the native environment of each inoculum (Table 2). NaCl was preferred over other salts exerting an impact on halophiles [13] as a compromise between inorganics in molasses and marine environments. Na^+^ and Cl^−^ are two of the five most concentrated inorganics in molasses (Additional file 1: Fig. S1), while NaCl provides most of the osmotic/salinity (about 90%) in marine standard media [39]. All enrichments were carried out in 3 independent biological replicates (i.e., 24 batch test reactors in total for each enrichment cycle). Controls with no substrate were prepared in duplicate at pH_i_ of 6 and 7 for both terrestrial and marine cultures (i.e., 8 negative controls in total for each enrichment cycle). Before starting the experiment, full anaerobic conditions were established in all serum bottles by flushing N_2_. Biogas production was evaluated through a water-displacement gas collection system to which the serum bottles were constantly connected. Water from the gas column was acidified to pH 3 to avoid CO_2_ absorption. Liquid and biogas sampling was conducted at day 0, 1, 2, 4, and 7.

### Continuously operated packed-bed biofilm reactors (PBBRs)

Following enrichments, 2 independent PBBRs were assembled for terrestrial- and marine-enriched cultures showing the highest accumulation of VFAs, i.e., OLR of 5 g_COD_ L^−1^ d^−1^ and pH_i_ 7. To operate PBBRs, the 3 independent biological replicates enriched at these conditions were pooled and used as inoculum for either the terrestrial or marine PBBR. These were constituted of a glass column where the carrier materials were placed, with a spherical decanter on the top for sludge and water separation (Additional file 1: Fig. S2). Working volumes were 1.7 and 1.8 L for terrestrial and marine cultures, respectively. PBBRs were loaded with polyvinyl carriers topped with a layer of ceramic carriers to avoid the packed-bed to float and placed in a temperature-controlled room at 34 °C. Recirculation of the liquid was set from bottom to top by a homemade peristaltic pump (Watson Marlow^®^ 323 pump head) providing an upflow velocity of 1.0–1.5 m h^−1^. pH control was set in the recirculation line and maintained at 7 by an automatic titration system with NaOH (Dulcometer^®^ D1Cb and DF2a pump, ProMinent^®^, Tubize, Belgium). Influents were introduced at the bottom of the PBBRs by a timer-controlled peristaltic pump (ProMinent^®^ DF2a, Belgium) that provided the daily flow required for each OLR. Effluent was withdrawn by overflow. Biogas was collected from the top of the decanter and its production was assessed through a water-displacement gas collection system to which PBBRs were constantly connected. Water from the gas columns was acidified to avoid CO_2_ absorption.

PBBRs were operated in batch mode for 7 days after mixing the inocula from enrichment tests with 35 g_COD_ L^−1^ of molasses to allow biomass formation on the packing material, and subsequently fed in continuous mode with a HRT equal to 10 days. The latter was selected to support carboxylate elongation, following observation of VFA production kinetics during the enrichments. In the first phase, PBBRs were operated at an OLR of 5 g_COD_ L^−1^ d^−1^ for 47 days. In a second phase, the OLR was increased to 10 g_COD_ L^−1^ d^−1^ and PBBRs were operated for another 30 days (for a total of about 2.5 months in continuous operation mode). Molasses were diluted 1:10 for the OLR 5 period and 1:5 for the OLR 10 period to facilitate influent pumping. Feed was kept mixed at 4 °C. As for the batch fermentation, salinity in the marine PBBR was increased by adding 22.8 g L^−1^ NaCl in the inlet, resulting in higher conductivity with respect to the terrestrial (Fig. 5a). Influents for OLR 10 presented more similar conductivities due to the proportional reduction in non-saline water used for dilution as compared to OLR 5. Liquid and biogas sampling from PBBRs was conducted every 2–3 days.

### Molecular analysis

#### DNA extraction

Samples (2 mL) from inocula and reactors were centrifuged in a FastPrep tube for 5 min at 13,000 rpm, and the pellets stored at −20 °C. Pellets were then thawed, and about 200 mg glass beads (0.11 mm, Sartorius) and 1000 μL lysis buffer (pH 8.0) were added. The tube was placed in a FastPrep device (MP Biomedicals, Santa Ana, USA) for two runs (16,000 rpm, 40 s) and centrifuged (10 min, max speed, 4 °C); the DNA was extracted from supernatants with phenol–chloroform and precipitated with 1 volume ice-cold isopropyl alcohol and 1:10 volume 3 M sodium acetate (1 h; −20 °C). Isopropyl alcohol was removed by centrifugation (30 min, max speed); DNA pellets were dried and resuspended in 30 µL 1× TE buffer (10 mM Tris, 1 mM EDTA) and immediately stored at −20 °C. The quality of DNA samples was assessed using 1% (w:v) agarose (Life technologies™, Madrid, Spain) gel electrophoresis, and DNA was quantified by a fluorescence assay (QuantiFluor^®^ dsDNA kit; Promega, Madison, USA) using a Glomax^®^-Multi + system (Promega, Madison, USA). Samples were then normalized to contain 1 ng µL^−1^ DNA and sent to LGC Genomics (Berlin, Germany) for library preparation and sequencing on an Illumina MiSeq platform. DNA amplification was conducted using the forward primer 341F 5′-NNNNNNNNNTCCTACGGGNGGCWGCAG and the reverse primer 785R 5′-NNNNNNNNNNTGACTACHVGGGTATCTAAKCC. For the sake of brevity, further details are provided in the Additional file 1: Supplementary Note S1.

#### DGGE

Following DNA extraction as reported above, PCR products were prepared using the forward primer PRBA338F-GC and the reverse 518R [40] at a concentration of 10 pmol μL^−1^ using the Fermentas kit (Thermo Scientific, Brussels, Belgium), applying 30 cycles. PCR products and DNA marker were supplied with about 4 μL loading dye, for a final volume of 10 μL per well. DGGE was prepared using Ingeny gel and kit (Goes, The Netherlands), final gradient being 45%, and run overnight at 120 V. Then, about 14 μL SYBR^®^ Green cell staining (concentrated 10^4^) was added to the gel and incubated for at least 20 min. Finally bands were visualized with an UV transilluminator equipped with SYBR^®^ Green filter.

### Chemical analysis

Total solids (TS), VS, volatile suspended solids (VSS), total Kjeldahl nitrogen (TKN), NH_4_^+^, and total COD were determined according to Standard Methods [41], while soluble COD (samples filtered at 0.45 μm) was analyzed using the Nanocolor^®^ kit (Macherey–Nagel, Germany). pH was measured using a pH probe (Herisau, Metrohm, Switzerland) and conductivity by a C833 conductivity meter (Consort, Turnhout, Belgium). Biogas quality composition was analyzed with a Compact GC (Global Analyser Solutions, Breda, The Netherlands), equipped with a Molsieve 5Å pre-column and two channels. In channel 1, a PoraBOND column detected CH_4_, O_2_, H_2_, and N_2_. In channel 2, an Rt-Q-bond pre-column and column detected CO_2_, N_2_O, and H_2_S. Biogas concentrations were determined with a thermal conductivity detector. Volatile fatty acids between C_2_ and C_8_ (including isoforms C_4_–C_6_) were measured by gas chromatography (GC-2014, Shimadzu^®^, The Netherlands) with DB-FFAP 123-3232 column (30 m × 0.32 mm × 0.25 µm; Agilent, Belgium) and a flame ionization detector (FID). Liquid samples were conditioned with sulfuric acid and sodium chloride and 2-methyl hexanoic acid as internal standard for quantification of further extraction with diethyl ether. Prepared sample (1 µL) was injected at 200 °C with a split ratio of 60 and a purge flow of 3 mL min^−1^. Oven temperature increased by 6 °C min^−1^ from 110 to 165 °C where it was kept for 2 min. FID had a temperature of 220 °C. The carrier gas was N_2_ at a flow rate of 2.49 mL min^−1^. Cations were measured by means of ion chromatography (IC, Metrohm IC 761, Herisau, Switzerland) with a Metrosep C6—250/4 column and Metrosep C4 183 Guard/4.0 guard column. A solution containing 1.7 mM HNO_3_ and 1.7 mM dipicolinic acid 184 was used as eluent. Anions were analyzed with the same equipment with a Metrosep A Supp 5–150 column and Metrosep A Supp 4/5 guard. A solution with 1 mM NaHCO_3_, 3.2 mM Na_2_CO_3_, and 5% (v:v) acetone was used as eluent.

### Calculations

COD conversion yields (COD to VFAs) were calculated as the amount of VFAs produced by reactors excluding the COD in the inlet already due to VFAs. This parameter indicated the degree of acidification (DA) according to the following equation:

$${\text{COD}}\;{\text{to}}\;{\text{VFAs}} = \frac{{{\text{VFA}}_{\text{OUT}} - {\text{VFA}}_{\text{IN}} \left[ {{\text{g}}_{\text{COD}} {\text{L}}^{ - 1} } \right]}}{{{\text{COD}}_{\text{IN}} - {\text{VFA}}_{\text{IN}} \left[ {{\text{g}}_{\text{COD}} {\text{L}}^{ - 1} } \right]}} \cdot 100.$$

VFAs bioconversion yields (*Y*_VFA_) were calculated according to [42] as

$$Y_{\text{VFA}} = \frac{{{\text{VFA}}_{\text{OUT}} - {\text{VFA}}_{\text{IN}} \left[ {{\text{g}}_{\text{COD}} {\text{L}}^{ - 1} } \right]}}{{({\text{COD}}_{\text{IN}} - {\text{VFA}}_{\text{IN}} ) - ({\text{COD}}_{\text{OUT}} - {\text{VFA}}_{\text{OUT}} ) \left[ {{\text{g}}_{\text{COD}} {\text{L}}^{ - 1} } \right]}} \cdot 100.$$

These two conversion yields differ as the *Y*_VFA_ does not consider the COD remaining in the effluent that was not converted to VFAs, thus resulting in slightly higher values as compared to DA. Methanogenesis yields were calculated as mL of CH_4_ produced per gram of COD removed, according to

$$Y_{{{\text{CH}}_{4} }} = \frac{{{\text{CH}}_{4} \left[ {{\text{mL}}\;{\text{L}}^{ - 1} \;{\text{d}}^{ - 1} } \right]}}{{{\text{COD}}_{\text{IN}} - {\text{COD}}_{\text{OUT}} \left[ {{\text{g}}_{\text{COD}} {\text{L}}^{ - 1} \;{\text{d}}^{ - 1} } \right]}} \cdot 100.$$

COD mass balances (COD tracked, Table 3) were calculated as the total amount of COD-equivalent VFAs, CH_4_, and biomass VSS daily production

$${\text{COD}}_{\text{tracked}} = \left( {{\text{VFA}}_{\text{OUT}} - {\text{VFA}}_{\text{IN}} } \right) + {\text{CH}}_{4} + {\text{VSS}} \left[ {{\text{g}}_{\text{COD}} {\text{L}}^{ - 1} \;{\text{d}}^{ - 1} } \right]$$

being the CH_4_ conversion factor to COD equal to 4 g_COD_/${\text{g}}_{{{\text{CH}}_{4} }}$, and the VSS to COD conversion factor equal to 1.2 g_COD_/g_VSS_ based on conservative measurements by [43].

**Table 3:** Final yields and productivities of molasses fermentation in terrestrial and marine PBBRs

PBBR	Fermentation					Methanogenesis			Biomass	COD balance			
	$\frac{\text{VFA}_{\text{IN}}}{{\text{COD}}_{\text{IN}}}$	$\frac{\text{VFA}_{\text{OUT}}}{{\text{COD}}_{\text{OUT}}}$	COD to VFAs	Y_VFA_	VFAs prod.	CH_4_ production		Yields	VSS	Fed	Removed	COD tracked	
					g_COD_ L^−1^ d^−1^	mL L^−1^ d^−1^	g_COD_ L^−1^ d^−1^	$\text{L}_{\text{CH}_{4}}$ g_COD_^−1^ removed	g_COD_ L^−1^ d^−1^	g_COD_ L^−1^ d^−1^		g_COD_ L^−1^ d^−1^	%
Terrestrial													
OLR 5													
*Mean*	*1.07*	*61.1*	*52.6*	*79.6*	*2.47*	*211.3*	*0.60*	*0.341*	*2.94*	*4.70*	*0.62*	*6.01*	*127.8*
SD	0.16	4.6	5.6	7.0	0.56	2.6	0.01		0.68	0.58	0.22		
OLR 10													
*Mean*	*1.38*	*74.7*	*66.7*	*85.8*	*6.77*	*362.7*	*1.04*	*0.324*	*3.40*	*9.82*	*1.12*	*11.21*	*114.2*
SD	0.96	9.6	9.3	8.0	3.15	2.4	0.01		1.17	3.42	0.79		

Marine													
OLR 5													
*Mean*	*0.95*	*60.8*	*59.5*	*102.3*	*2.36*	*114.9*	*0.33*	*0.280*	*2.33*	*4.90*	*0.41*	*5.02*	*102.5*
SD	0.23	7.7	7.2	27.5	0.56	8.0	0.02		0.62	2.14	0.32		
OLR 10													
*Mean*	*1.20*	*66.8*	*60.3*	*87.8*	*5.89*	*299.3*	*0.86*	*0.374*	*3.61*	*9.80*	*0.80*	*10.36*	*105.7*

Mean and standard deviations refer to the last 10 days of operation. Conversion yields were assessed as indicated in *Calculations*, in “Methods” section. VSS in the effluent were considered as estimates of biomass production. *Y*_VFA_ is typically higher than COD to VFAs yield as in the former the COD in the outlet not converted to VFAs is not considered. While the former is an indication of the specific microbial activity, the COD to VFAs yield is a process parameter

Standard Gibbs energy change (Δ*G* ^0^) was calculated from the Gibbs free energy of formation (*ΔG*_f_^0^) values of the compounds participating in the reaction [44] and the stoichiometry of the reaction. Compensation by temperature ($\Delta G^{01}_{{35^\circ {\text{C}}}}$) was calculated using the Gibbs–Helmholtz equation and correction at biological standard state (pH 7 and 6, $\Delta G^{01}_{{35^\circ {\text{C}}}}$) as reported in [45].

### Statistical analysis

In batch tests, results are expressed as mean values of experiments made in 3 independent replicates, with error bars indicating standard deviation from the mean. In PBBR experiments, mean values indicate the last phase of operation when stable conditions were achieved (last 10 days), with error indicating standard deviation from the mean. Where indicated, statistical significance was assessed using a non-parametric test (Mann–Whitney test) that considered a two-sided distribution with 95% confidence interval. Analysis of the 16S rRNA gene DGGE was performed using BioNumerics (Applied Maths, version 5.1), which assigned band classes. Fuzzy clustering was performed using the Jaccard distance (aware of band intensity). Statistical analyses were performed using the R language for statistical programming (version 3.3.0). Ecological analyses (ordination and rarefaction curves) were performed using the R package vegan, version 2.3–5 [46] on the common-scaled OTU table (Additional file 1: Supplementary Note S1).

## Results and discussion

### Molasses fermentation turns into a brine treatment process

Fermentation of molasses into VFAs was performed using marine sediment or anaerobic digestion sludge to test whether adaptation to salinity in marine microbes enhances molasses bioconversion efficiency. Microbial cultures were initially enriched in batch and maintained in their respective salinity range (native conductivity equivalent to 1.6 and 19.1 mS cm^−1^ in terrestrial and marine inocula, respectively, Table 2).

**Fig. 1:**
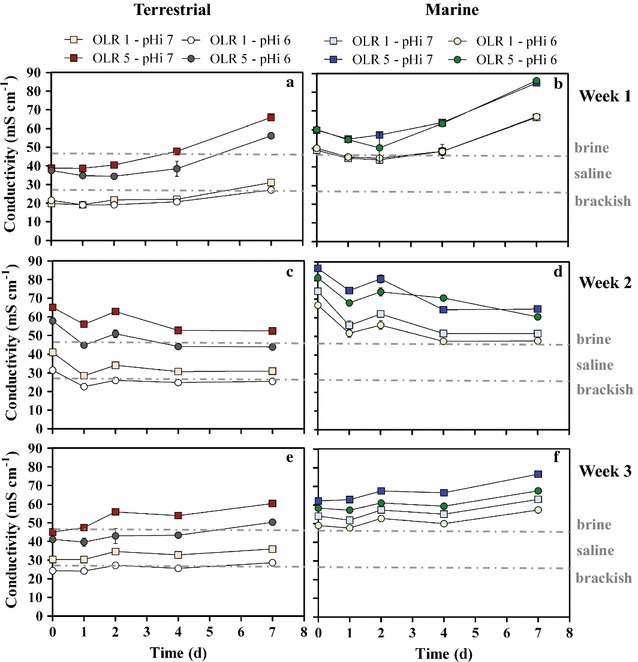
Conductivity values for terrestrial (**a**, **c**,** e**) and marine (**b**, **d**, **f**) cultures during enrichments. *Dotted gray line* indicates the nominal change of environment from brackish to saline, to brine waters. *Weeks 1* (**a** and **b**), *2* (**c** and **d**), and *3* (**e** and **f**) indicate the sequential fermentation batches that constituted the enrichment. Mean values are the average of experiments done in three independent replicates. *Error bars* indicate standard deviation from the mean. Keys reported in the graph

Feeding molasses with a high ionic composition (Additional file 1: Fig. S1) resulted into higher conductivities as compared to those normally experienced by both terrestrial and marine environments (Table 1), resembling a brine treatment process. In terrestrial cultures, molasses dilution in tap water determined an initial conductivity of about 20 and 40 mS cm^−1^ (pH_i_ 6 and 7, respectively, Fig. 1a). Nonetheless the conductivity of raw molasses, a highly viscous syrup with conductivities corresponding to brackish-saline waters, was only 29 ± 5 mS cm^−1^ (Table 2). Raw molasses dilution 1:4 with tap water resulted in higher conductivities (i.e., 52 mS cm^−1^), with formation of a gray precipitate and clarification of the liquid phase. An increase in conductivity in the present batch of diluted molasses was observed up to a ratio 1:10 (De Vrieze J., personal communication). This phenomenon was explained as a reduction of viscosity or particles solubilization in water that allows ions to be set free in solution and increase their electrical conductivity potential [47]. Initial conductivity in marine cultures resembled brine waters (>47 mS cm^−1^, Fig. 1; Table 1), while terrestrial cultivation conditions spanned between brackish and saline during enrichment tests (20–47 mS cm^−1^, Fig. 1; Table 1). Repeated inoculum transfer along the enrichments resulted in increased conductivities, leading to brine environments for the highest organic load tested with terrestrial cultures (52.2 ± 9.2 mS cm^−1^, Fig. 1e) and much higher values for the correspondent marine (68.8 ± 10.0 mS cm^−1^, Fig. 1f) (*p* < 0.001).

Conductivity was also impacted by VFA production, as indicated by the increasing profile in the first week of fermentation (up to 25 mS cm^−1^ higher at day 7, Fig. 1a, b). The latter is explained by an acid–base equilibrium. The majority of the detected VFAs have a pKa around 4.8, thus VFA production is balanced via NaOH addition. Base addition is used as pH stabilization and counter ion for the anionic VFA fraction. At pH 7, about 99% of the VFAs are present in the anionic form while at pH 6 about 5–6% of the total shift to the acid form which, together with a lesser base addition, slightly reduces conductivity (Fig. 1). Finally, high OLRs resulted in high VFA production, which further increased conductivities when comparing OLR 1 and OLR 5.

### Comparable molasses fermentation by diverse enrichments originated from terrestrial and marine inocula

#### Biogas production

**Fig. 2:**
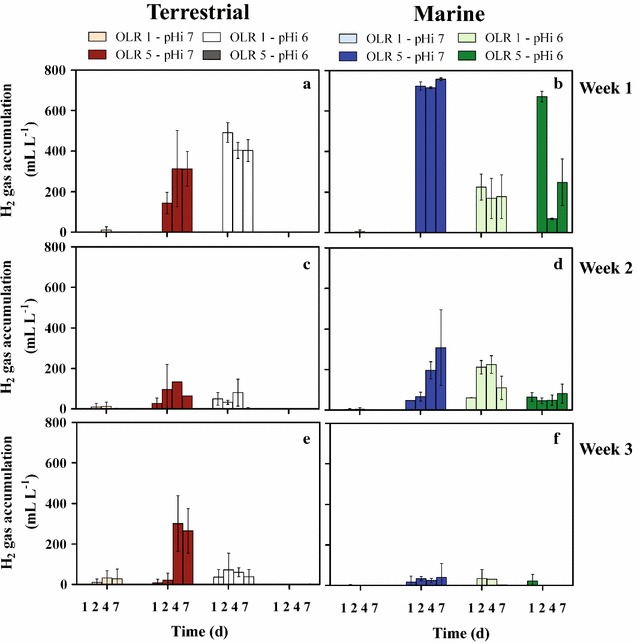
H_2_ gas accumulation during the enrichment of terrestrial (**a**, **c**, **e**) and marine (**b**, **d**, **f**) cultures using molasses. Cultures were tested in batch and had different initial pH values (pHi) (either 6 or 7) and organic loading rates (OLR) (either 1 or 5 g_COD_ L^−1^ d^−1^, equivalent to an initial content of 7 or 35 g_COD_ L^−1^, respectively). Temperature was set to 35 °C. Cultures were tested for 7 days, after which 10% liquid volume was withdrawn and incubated again with fresh medium for another 7 days. Hence, the enrichment consisted of 3 consecutive batches of 1 week each. Marine cultures were provided with 23 g L^−1^ NaCl to maintain their original salinity in all conditions. *Error bars* represent standard deviations of 3 independent biological replicates. Keys reported in the graph

Biogas production varied widely in terrestrial and marine cultures during the enrichment, mainly due to H_2_ production. In anaerobic digestion, reduction of the substrate contact time [48] or overloading of the system [49] may cause accumulation of intermediate compounds (e.g., H_2_). During the first hydrolytic and acidogenic steps of anaerobic digestion, the high activity by H_2_-producing species is not counterbalanced by that of other microbial representatives which continue the fermentation process, leading to H_2_ accumulation. This becomes evident in sub-optimal conditions when high concentrations of readily available organics (i.e., sugars) are fed to communities non-adapted to such organics (i.e., marine) or not well acclimated (terrestrial). In the latter, H_2_ gas was produced only at OLR 5—pH_i_ 7 and at OLR 1—pH_i_ 6 (Fig. 2a). The H_2_ production profile suggested a feeding overload at pH_i_ 7, with productivity maintained throughout the enrichment only at OLR 5—pH_i_ 7 (to 301 ± 137 mL L^−1^, Fig. 2a, c, e). On the contrary, the H_2_ production profile suggests an inhibiting effect at pH_i_ 6 and OLR 5, with acclimation to molasses bioprocessing at low OLR and pH_i_ 6 eventually circumventing H_2_ accumulation (Fig. 2b, d, f). Concerning marine cultures, they were initially very productive towards H_2_ gas at all conditions except for OLR 1—pH_i_ 7, the mildest conditions, with the highest values recorded at OLR 5—pH_i_ 7 (757 ± 7 mL L^−1^, Fig. 2b). This observation supports the hypothesis that high degradation rates of readily available organics were uncommon to marine microbes. Nonetheless, acclimation to sugar degradation at the end of week 3 resulted in almost no H_2_ gas production in any marine culture (Fig. 2f).

**Fig. 3:**
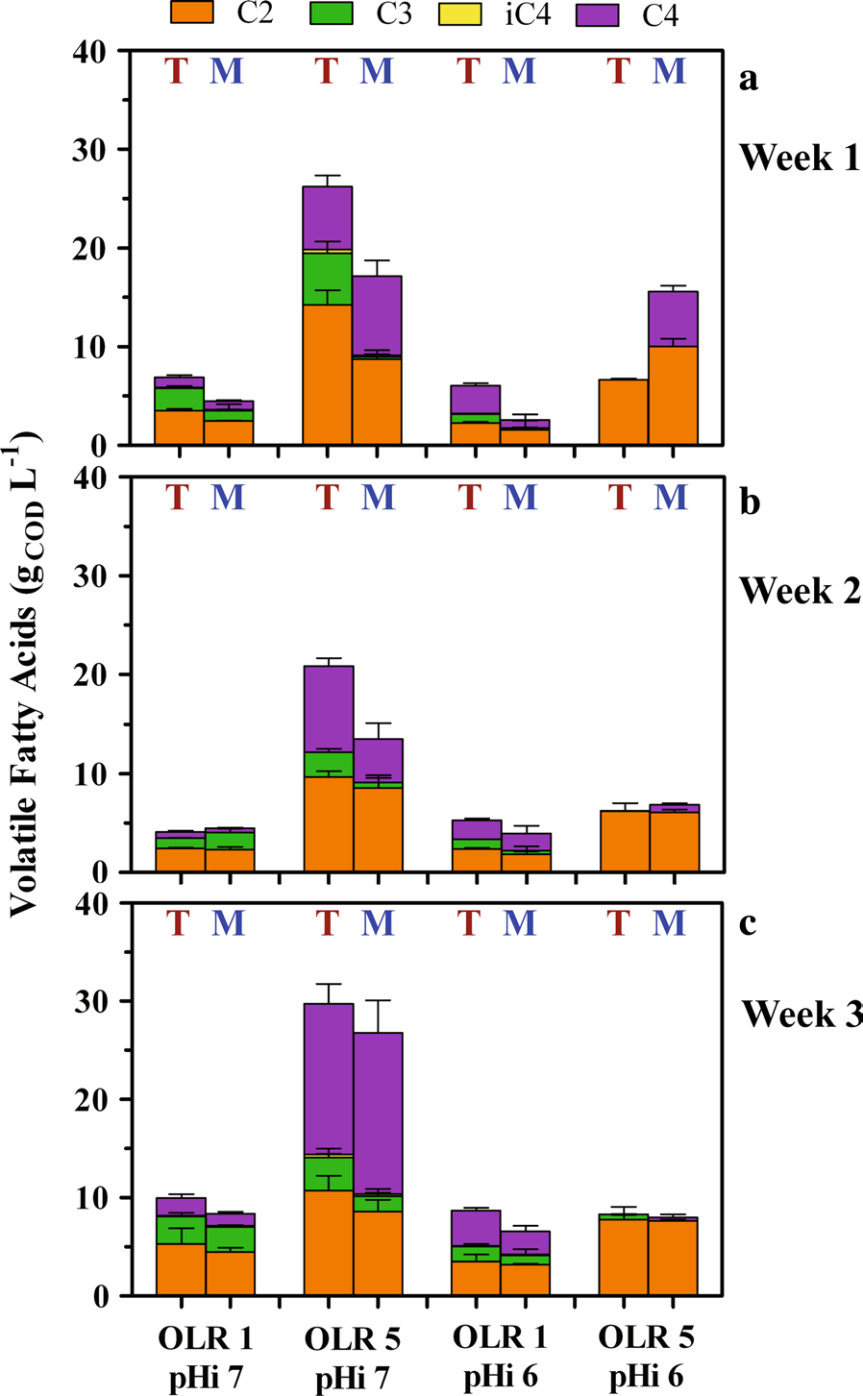
Final volatile fatty acids (VFAs) accumulation during the enrichment of terrestrial (T) and marine (M) cultures using molasses. Final values refer VFAs concentration at the last day of incubation (day 7). Cultures were tested in batch and had different initial pH values (pHi) (either 6 or 7) and organic loading rates (OLR) (either 1 or 5 g_COD_ L^−1^ d^−1^, equivalent to an initial content of 7 or 35 g_COD_ L^−1^, respectively). Cultures were tested for 7 days, after which 10% liquid volume was withdrawn and incubated again with fresh medium for another 7 days. Hence, the enrichment consisted of 3 consecutive batches of 1 week each (*Week 1*, **a**; *Week 2*, **b**; *Week 3*, **c**). Marine cultures were provided with 23 g L^−1^ NaCl to maintain their original salinity at all conditions. *Error bars* represent standard deviations of 3 independent biological replicates. Keys reported in the graph

CO_2_ production along the enrichment was generally low and constant irrespective of pH_i_ and inoculum origin when OLR was set to 1 g_COD_ L^−1^ d^−1^ (Additional file 1: Fig. S3). The most remarkable changes were observed at OLR 5—pH_i_ 7, where CO_2_ production in terrestrial cultures dropped from 961 ± 69 mL L^−1^ in week 1 to 187 ± 166 mL L^−1^ in week 3 (Additional file 1: Fig. S3A and E, respectively) while the opposite was true in marine reactors (from 396 ± 357 to 1060 ± 305 mL L^−1^, week 1 and 3, respectively, Additional file 1: Fig. S3B and F). This was explained as an acclimation of the terrestrial inoculum and an increase of activity in the marine, corroborated by constant VFA production in the former and an increase in the latter (Fig. 3). As a result, at OLR 5—pH_i_ 7 a very different H_2_:CO_2_ ratio was noted, with terrestrial cultures maintaining values ~0.5 during the whole enrichment period, contrary to marine where the ratio dropped from 1.9 ± 0.8 to 0.06 ± 0.07 (week 1–3, Additional file 1: Fig. S4). CH_4_ was never detected during the whole enrichment phase in any batch test (detection limit 0.01%).

#### VFAs production

Biogas kinetics were consistent with the acclimation to molasses fermentation and may be descriptive of the pathways for sugar conversion into VFAs. In all the H_2_-producing batch reactors the highest accumulation peak was not observed before day 2, together with butyrate, while acetate was produced at high levels from the beginning of the incubation (Additional file 1: Fig. S5). The pathways describing such fermentation kinetics were considered with respect to their standard Gibbs energy change (Δ*G* ^0^), compensated by temperature and corrected for their biological standard state (pH 7 or pH 6, $\Delta G^{01}_{{35^\circ {\text{C}}}}$). One of the most applied bioconversion equations from sugar comprises ethanol production (Eq. 1), followed by oxidation to acetate and subsequent H_2_ generation (Eq. 2):

1 $${\text{C}}_{6} {\text{H}}_{12} {\text{O}}_{6} \to 2{\text{CH}}_{3} {\text{CH}}_{2} {\text{OH}} + 2{\text{CO}}_{2} \Rightarrow \Delta G_{{35{^\circ }{\text{C}}}}^{01} = - 233.5\;{\text{kJ}}\;{\text{mol}}^{ - 1} \left( {{\text{pH}}\;6\;{\text{and}}\;7} \right)$$

2 $$\begin{aligned} {\text{CH}}_{3} {\text{CH}}_{2} {\text{OH}} + {\text{H}}_{2} {\text{O}} \to {\text{CH}}_{3} {\text{COOH}} + 2{\text{H}}_{2} \Rightarrow \Delta G_{{35{^\circ }{\text{C}}}}^{01} & = + 8.6\;{\text{kJ}}\;{\text{mol}}^{ - 1} \left( {{\text{pH}}\;7} \right) \\ \Rightarrow \, \Delta G_{{35{^\circ }{\text{C}}}}^{01} & = + 14.3\;{\text{kJ}}\;{\text{mol}}^{ - 1} \left( {{\text{pH}}\;6} \right) \\ \end{aligned}$$

Provided that H_2_ production only occurs after ethanol has been accumulated, thus requiring longer retention times, this pathway would be consistent with H_2_ accumulation observed at day 2 (Fig. 2) but would not explain acetate accumulation already at day 1 (Additional file 1: Fig. S5). Alternatively, H_2_ (and CO_2_) may derive from glucose oxidation to butyrate (Eq. 3), consistent with butyrate accumulation also occurring after day 2 (Additional file 1: Fig. S5):

3 $$\begin{aligned} {\text{C}}_{6} {\text{H}}_{12} {\text{O}}_{6} \to {\text{CH}}_{3} \left( {{\text{CH}}_{2} } \right)_{2} {\text{COOH}} + 2{\text{CO}}_{2} + 2{\text{H}}_{2} \Rightarrow \Delta G_{{35{^\circ }{\text{C}}}}^{01} & = - 277.2\;{\text{kJ}}\;{\text{mol}}^{ - 1} \left( {{\text{pH }}7} \right) \\ \Rightarrow \Delta G_{{35{^\circ }{\text{C}}}}^{01} & = - 271.5\;{\text{kJ}}\;{\text{mol}}^{ - 1} \left( {{\text{pH}}\; 6} \right) \\ \end{aligned}.$$

Acetate accumulation may potentially derive directly from glucose as described in Eq. 4.

4 $$\begin{aligned} {\text{C}}_{6} {\text{H}}_{12} {\text{O}}_{6} \to 3{\text{CH}}_{3} {\text{COOH}} \Rightarrow \Delta G_{{35{^\circ }{\text{C}}}}^{01} & = - 318.4\;{\text{kJ}}\;{\text{mol}}^{ - 1} \left( {{\text{pH}}\;7} \right) \\ \Rightarrow \Delta G_{{35{^\circ }{\text{C}}}}^{01} & = - 301.3\;{\text{kJ}}\;{\text{mol}}^{ - 1} \left( {{\text{pH}}\;6} \right) \\ \end{aligned}$$

Comparison of the $\Delta G^{01}_{{35^\circ {\text{C}}}}$ between Eqs. 1 and 2 as opposed to Eqs. 3 and 4 suggests that the latter may have been favored over the former at both pH conditions; however, the possibility that all these pathways operated concomitantly or compensated each other to some extent cannot be ruled out.

The enrichment procedure increased the biological activity of all cultures, which started to accumulate higher VFA levels at day 1 when comparing week 1 versus week 2 and 3 in both terrestrial and marine systems (Additional file 1: Fig. S6). This was mirrored in the pH pattern, where shifts from either 6 or 7 occurred sooner in the 7-day incubation period already at week 2 (Additional file 1: Fig. S7). Differences in VFA productivity observed at week 1 at each pH condition were leveled off at the end of the enrichment (week 3, day 7), as marine and terrestrial cultures had comparable biochemical activities with a maximum VFA production of about 30 g_COD_ L^−1^ at pH_i_ 7 and OLR 5 (Fig. 3c). Similarly, the VFA composition profile converged at optimal conditions (OLR 5—pH_i_ 7) to about 33% acetic, 8% propionic, and 55% butyric acid. There was no substantial impact on longer carboxylates due to the inoculum origin, enrichment, or OLR (*p* > 0.05), as in all conditions VFAs accumulated consistently only up to C4 (Fig. 3), with the sum of all the final VFA concentrations between C5 and C6 not higher than 0.66 ± 0.37 g_COD_ L^−1^ (observed in terrestrial cultures at OLR 5—pH_i_ 7). This supports the hypothesis that the pathways described in Eqs. 3 and 4 were favored over those in Eqs. 1 and 2, as the lack of reducing power (e.g., ethanol from Eq. 1) would impair carboxylate elongation [35].

#### Microbial communities

**Fig. 4:**
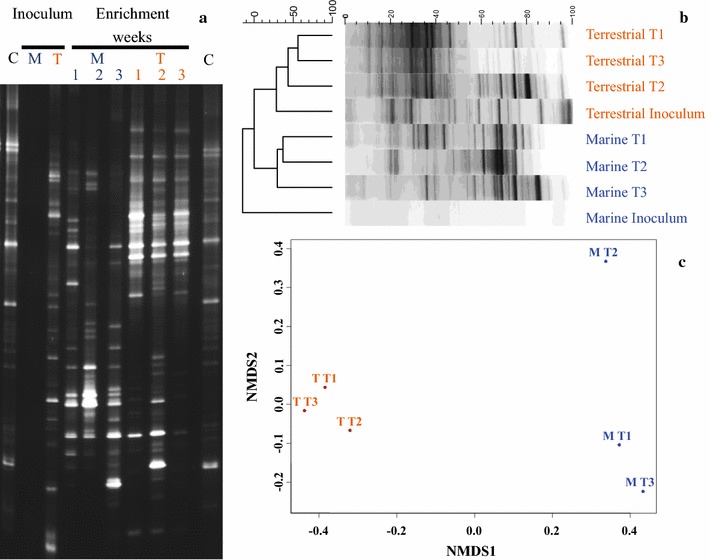
DGGE profiles (**a**), Jaccard matrix (**b**), and NMDS analysis (**c**) of terrestrial (T) and marine (M) inocula and cultures enriched at OLR 5—pH_i_ 7. Samples for inocula are representative of cultures prior to any enrichment with molasses. DNA extraction from molasses did not yield any result (data not shown). Samples were collected at the end of the 7-day incubation period for each of the 3 weeks of the enrichment. Fuzzy clustering was performed using the Jaccard distance (aware of band intensity), while community structure (relative abundances) analysis used the abundance-based Jaccard dissimilarity index. Samples closer to one another have a more comparable community structure. NMDS1 shows a clear separation between terrestrial and marine cultures along the enrichment

Employing a different initial inoculum had an impact on the final microbial community structure (Fig. 4). Few DNA bands in the terrestrial inoculum were maintained in the enriched consortia, while the high richness and evenness typical of marine sediments [50–52] resulted in no visible DNA band for the inoculum (Fig. 4a), despite yielding DNA during the extraction (as assessed by agarose gel electrophoresis, data not shown). Non-metric multidimensional scaling (NMDS) analysis of the enriching microbial communities indicated that marine and terrestrial cultures evolved through different pathways (Fig. 4b, c). Provided that DNA extraction from molasses diluted 1:10 was negative, no substantial contribution of microbes due to the feedstock occurred. The common microbial activity between terrestrial and marine communities did not result from a massive contamination by microbes in the substrate, rather from similar biochemical reactions carried out by different cultures.

### Highly efficient, continuously operated PBBRs convert molasses into VFAs at brine salinities

#### Continuously operated brine systems

**Fig. 5:**
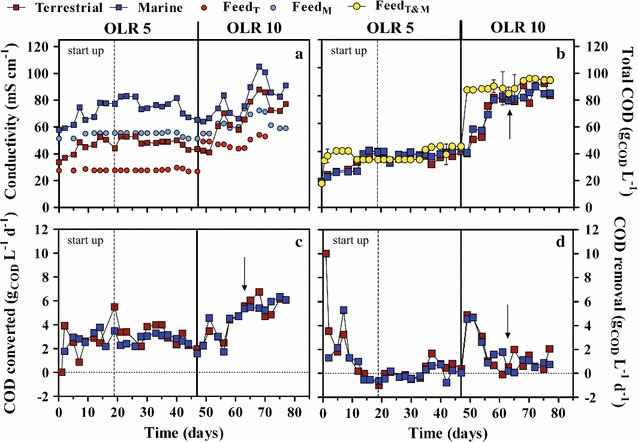
Conductivity (**a**), total COD (**b**), and COD conversion (**c**) and removal (**d**) rates in terrestrial and marine PBBRs. Terrestrial (T) and marine (M) PBBRs were operated at a controlled pH equal to 7, temperature was set to 34 °C. Following 7 days of recirculation to allow biomass development in the PBBRs, both reactors were continuously fed with molasses with a hydraulic retention time (HRT) of 10 days. Time zero represents the first day that PBBRs were operated continuously. Hence, the startup period refers to almost entire 2 retention times (day 19) where PBBRs were continuously fed before observing constant productivity in VFAs and other related parameters. PBBRs were fed with an OLR equal to 5 g L^−1^ d^−1^ until day 47, after which the OLR was increased to 10 g L^−1^ d^−1^ by reducing the dilution rate of molasses. Operational parameters reached a renewed stability at day 63, although this did not apply to conductivities values (**a**). Marine cultures were provided with 23 g L^−1^ NaCl to maintain a higher salinity with respect to terrestrial cultures. *Error bars* only refer to the influent COD (Feed_T&M_) and represent standard deviations of 2 independent replicates. Keys reported in the graph

Enriched terrestrial and marine cultures at OLR 5—pH_i_ 7 yielding the highest accumulation of VFAs (Fig. 3c) were loaded into two PBBRs for continuous operation at these same culture conditions. The HRT was extended from 7 to 10 days to support the accumulation of longer carboxylates beyond C4 [35]. As for the enrichments, molasses fed to marine cultures was supplied with ~23 g L^−1^ NaCl resulting in conductivities in the inlet typical of brine waters (58 mS cm^−1^), while the terrestrial PBBR inlet had lower conductivities values (33 mS cm^−1^) typical of saline waters (Fig. 5a; Table 1). This divergence was maintained as long as the OLR was set to 5 g_COD_ L^−1^ d^−1^ (i.e., day 47, Fig. 5a). Conductivity was similar in inlet and outlet of each PBBR at the start up of continuous operation (0 days, Fig. 5a) due to the previous batch mode of operation allowing biomass formation on the packing material and, consistent with batch tests, it increased in outlets until stable VFA production was reached. Carboxylates were all present at 99% in their ionic form as pH was continuously controlled at 7 (pKa of VFA 4.8). The difference in conductivity between inlet and outlet due to the accumulated VFAs accounted for 17 and 24 mS cm^−1^ for terrestrial and marine PBBR, respectively, resembling batch tests results (Fig. 1).

Conductivity divergence between the two systems was later reduced when the OLR was doubled (day 47, OLR 10, Fig. 5a) owing to a reduced molasses dilution in the inlet which supplied more salts to PBBRs. Conductivities in both inlets were kept >50 mS cm^−1^ (from day 55, Fig. 5a) turning molasses fermentation into a brine treatment process, with VFA production further increasing outlets conductivity to about 8 and 23 mS cm^−1^ in terrestrial and marine PBBR, respectively.

The difference in salinity had no impact on the inlet COD and both PBBRs were fed with equal amounts of organic matter (Fig. 5b). Due to molasses density, the actual COD fed to both PBBRs was slightly lower than 5 and 10 g_COD_ L^−1^ d^−1^ (Table 3), although reproducible along the experiment.

In terms of organic bioconversion, continuous operation reached stability after less than 20 days (start up, dotted line, Fig. 5) as indicated by the COD pattern in inlet and outlets (Fig. 5b). According to mass balances, concentrations should equalize after one full HRT (i.e., 10 days). However, a sustained COD removal rate during the initial operation (Fig. 5d) increased the hydraulic stabilization time, likely due to bacteria acclimating to the continuous feeding. This trend was mirrored at the beginning of OLR 10 (day 47, straight line, Fig. 5), when cultures had to acclimate to the increase in both salinity and carbon input (Fig. 5b, d) and eventually stabilized at day 63 (arrows, Fig. 5), confirming an almost doubled theoretical stabilization time. Acclimation to new operational conditions affected COD conversion rates, which increased and stabilized after ~18 days following a change in operational parameters (from day 18, OLR 5; from day 63, OLR 10; Fig. 5c). However, despite the difference in conductivity in terrestrial and marine cultures, particularly at OLR 5, total COD, COD removal, and conversion rates were comparable in the two PBBRs (Fig. 5b–d) (*p* > 0.05).

#### Biogas production

**Fig. 6:**
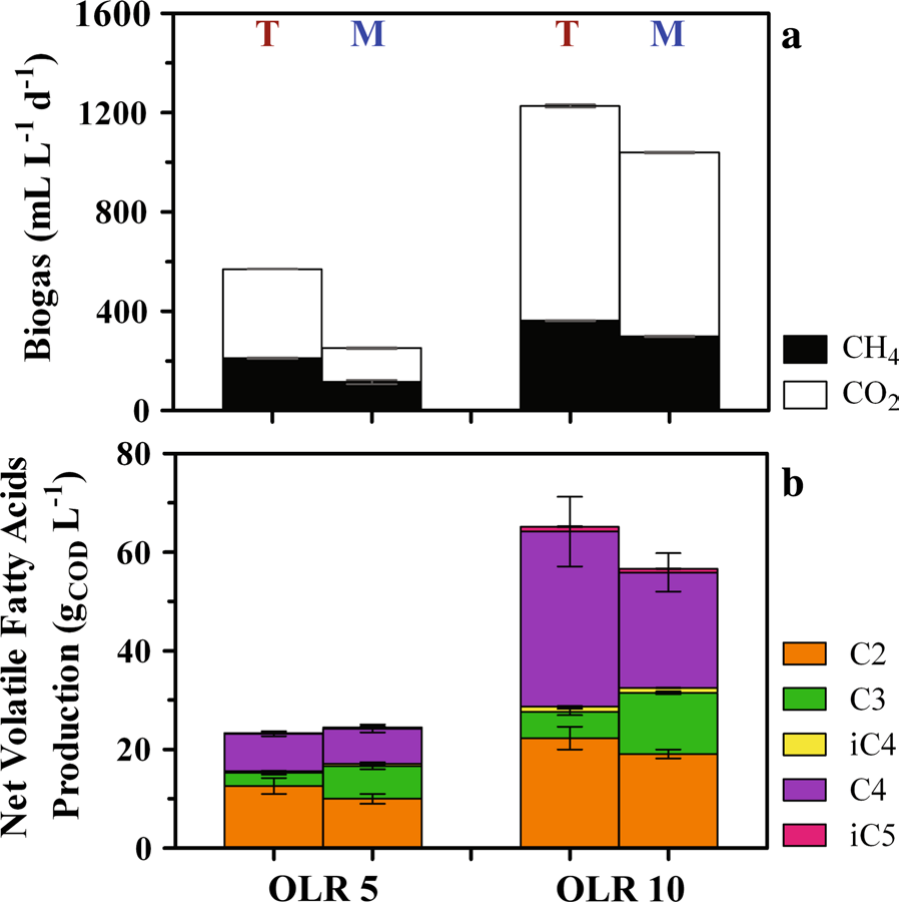
Biogas (**a**) and net VFAs productivity (**b**) in terrestrial and marine PBBRs. All reactors were operated at pH 7, with a HRT of 10 days, temperature equal to 34 °C. For both terrestrial (T) and marine (M) PBBRs, mean and standard deviations refer to the last 10 days of operation. Keys reported in the graph

The main result of extending the HRT to 10 days as compared to batch tests was CH_4_ production, which was about 211 ± 3 and 115 ± 8 mL L^−1^ d^−1^ at OLR 5 in terrestrial and marine PBBRs, respectively (Fig. 6a). These accounted only for about 13 and 7% of the total fed COD that was removed as CH_4_ gas, but presented an exceptional conversion rate compared to the theoretical yield of 0.35 L CH_4_ g_COD_^−1^ removed (Table 3). Consistently, H_2_ gas was only detected at low titers during the start up phase (day 0–18) and never thereafter (detection limit 0.01%). At OLR 5, the terrestrial PBBR produced also more CO_2_ (359 ± 1 vs. 137 ± 4 mL L^−1^ d^−1^ in the marine PBBR, Fig. 6a), which resulted in a lower CH_4_ relative content in the total produced biogas as compared to the marine PBBR (37 ± 1 vs. 46 ± 3%). Doubling the OLR to 10 g_COD_ L^−1^ d^−1^ resulted in a remarkable increase in biogas production to 1.2 and 1 L L^−1^ d^−1^ (total biogas in terrestrial and marine PBBRs, respectively, Fig. 6a), with the relative CH_4_ content leveling down to ~29% in both systems. CH_4_ production may result from hydrogenotrophic methanogens coupling CO_2_ and H_2_, or by acetoclastic methanogens solely relying on acetate [53]. Provided that H_2_ gas was not detected and that hydrogenotrophic methanogens are more resistant than acetoclastic to high conductivities [28], CH_4_ formation in the present systems may derive from hydrogenotrophic communities.

#### VFAs production

The extended HRT to 10 days did not yield much carboxylates beyond C4 as compared to the enrichments, as isovaleric (but not valeric) acid was never found at concentrations higher than 1 g_COD_ L^−1^ even at OLR 10 (Fig. 6b). Together with previous observations [28, 34], batch test enrichments and their potential pathways (Eqs. 3, 4), this result supports the hypothesis that molasses is unsuitable for consistent production of carboxylates longer than C4. Net VFA productivity was comparable in terrestrial and marine PBBRs at both OLR 5 and 10 (*p* > 0.05, Fig. 6b), although propionic acid was more pronouncedly produced in the marine at both OLRs (*p* < 0.05; Fig. 6b). Doubling the OLR resulted in a proportional increase in VFA concentration, which reached 60–65 g_COD_ L^−1^ in both culture systems (Fig. 6b). These were equivalent to remarkably high yields in terms of specific microbial activity (*Y*_VFA_ ≥ 85%, Table 3) and molasses processing (COD to VFAs up to 66.7 ± 9.3%, Table 3). The latter is comparable to the highest observed for fermentation over a wide range of agro-industrial leftovers (Table 4) that, contrary to the present study, were obtained by operating under low salinity. At stable operational conditions during OLR 10 (after day 63, Fig. 5b–d) the marine PBBR was operated at 90.2 ± 8.0 mS cm^−1^ (Fig. 5a). This value is 3 times higher than that inhibiting methanogenesis (30 mS cm^−1^) and inducing a wash out in upflow anaerobic sludge blanket (UASB) reactors operated with granular sludge at a HRT of 3 days using the same batch of diluted molasses as in the present study [28]. PBBRs adopted in this investigation allowed for a better VFA conversion at higher conductivities and, probably due to a longer HRT, a higher CH_4_ production. This postulates that further improvements in VFA yields may be expected by reducing HRTs, thereby preventing methanogenesis.

**Table 4:** Comparison of acidogenic digestion yields using different agro-industrial leftovers

Biowaste origin	OLR (g_COD_ L^−1^ d^−1^)	Max COD to VFAs (%)	Reference	Notes
Molasses	9.81 ± 0.1	63.5 ± 3.2	This study	Average of marine and terrestrial PBBRs
Dairy	8.0	48.4	[69]	Includes alcohols
Olive mill	13.3	66.0	[70]	Used a PBBR
Dairy	4.0	59.1	[71]	Includes alcohols
Food	83.3	43.4	[72]	Lower OLR (44 g_COD_ L^−1^ d^−1^) had also lower efficiencies (30.5%)
Sugar beet	2.7	64.2	[34]	Chemicals addition

#### COD mass balance

An overall balance of COD conversion into VFAs, CH_4_, and biomass was calculated for all PBBRs covering around 100% of the total COD fed (Table 3), with estimates on the biomass production calculated using the VSS content present in the effluents. The latter accounted for ~30% of the converted COD, and was 3–5 times higher than the COD converted into CH_4_. Continuous generation of biomass in PBBRs, rather than methanogenic activity, may explain the slightly reduced performance in net VFAs generation (Fig. 6b) as compared to batch systems (Fig. 3c) when operating under the same conditions (OLR 5—pH_i_ 7). During batch tests, cultures at OLR 5 had 10–12 g_VSS_ L^−1^, irrespective of pH_i_ and inoculum (Additional file 1: Fig. S8). These values are consistent with those measured in continuously operated PBBRs at OLR 5 (11.9 ± 3.2 and 11.7 ± 3.1 g_VSS_ L^−1^, in terrestrial and marine PBBR), which increased ~40% by doubling the COD in the inlet at OLR 10. While not resulting in PBBRs wash out, continuous operation in highly saline systems increased the amount of energy dedicated to microbial biomass formation, the optimization of which may further improve molasses bioconversion into VFAs.

### Culture conditions select for a shared functionality in diverse microbial communities originating from diverse environments

#### Microbial community structure

**Fig. 7:**
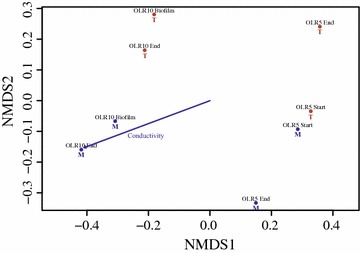
NMDS analysis of microbial community variance in terrestrial and marine PBBRs. Community structure (relative abundances) analysis using the abundance-based Jaccard dissimilarity index on common-scaled data after removing singletons. Samples closer to one another have a more comparable community structure. NMDS 1 shows a clear separation depending upon OLR (either 5 or 10 g L^−1^ d^−1^), while NMDS 2 shows separation upon PBBR source (either terrestrial [T] or marine [M]). The stress (inertia) for the plot is 0.041, with a non-metric *R* ^2^ equal to 0.998. A statistically significant correlation (*p* = 0.02697, 1000 permutations) explains the separation between terrestrial and marine PBBR samples

The central hypothesis of the present study was that salinity is potentially a driving factor in molasses fermentation, with marine communities having an advantage over terrestrial in converting sugars to VFAs. On the other hand, high sugar concentrations are uncommon to marine microbial communities, contrary to terrestrial from anaerobic digesters. While maintaining a different composition (Fig. 7, Additional file 1: Fig. S9), microbial community structures evolved through different mechanisms to an equivalent fermentation efficiency (Fig. 6b; Table 3). The same conclusion was achieved using different cultivation systems and molecular techniques during the enrichment (Fig. 4). This suggests that culture conditions selected for specific microbial functionalities rather than representatives or, alternatively, that equivalent pathways were present in both environments, although supported by different genera.

Salinity and OLR had an impact in microbial community structures. Greater differences in conductivities between PBBRs operated at OLR 5 as compared to OLR 10 (Fig. 6a) were reflected in a greater dissimilarity between microbial communities (Fig. 7), with conductivity being significantly correlated with marine PBBRs (*p* = 0.02697). The increase in OLRs markedly influenced the community composition and moderately decreased the dissimilarity between terrestrial and marine PBBRs, with samples derived from effluents (OLR 10 End, Fig. 7) and biofilms (OLR 10 Biofilm, Fig. 7) not being dissimilar in either PBBR. Community diversity was higher at OLR 10 than at OLR 5 (Additional file 1: Fig. S10) and, notwithstanding the comparable biochemical response (Fig. 6), marine communities were more diverse than terrestrial (Additional file 1: Fig. S9). In conclusion, terrestrial microbial cultures non-acclimated to salinity can be enriched to attain high-performing molasses fermentation. However, marine cultures may be better at tolerating salinity shocks and other stressful conditions during continuous processes, as high genetic diversities are often related with communities possessing greater options to face environmental changes [54, 55].

#### Enriched microbial representatives

Alongside sugar conversion to VFAs, a shared functionality in efficient PBBRs operating at high salinity (about 90 mS cm^−1^) was biomass turnover (Table 3). Bacterial sequences common to both PBBRs at OLR 10 and whose abundance was enriched >1% of the total microbial community (Additional file 1: Table S1) included an unclassified *Aminobacterium* (OTU0005), *Bacteroidetes coprosuis* (OTU0002, OTU0003, and OTU0004), an unclassified *Tepidimicrobium* (OTU0007), and an unclassified *Clostridiales*, tentatively identified as *Tissierella Soehngenia* (OTU0009) (complete list of all bacterial sequences is reported in Additional file 2: Table S2).

The *Aminobacterium* genus was first proposed as a salt-tolerating, amino acid (rather than carbohydrate) fermenter, H_2_ and acetate producer, inhabiting anaerobic sludges of diary wastewater treatment plants [56]. It was positively associated with increasing OLRs in UASB reactors digesting molasses wastewaters, but negatively with CH_4_ production [57]. *Tepidimicrobium* was first introduced as a thermophilic, protein-degrading genus [58], which is positively associated with high VFA concentrations in reactors co-digesting molasses with other bioresidues [59]. *B. coprosuis* was first isolated from swine manure as a mesophilic, anaerobic microbe which ferments glucose to acetate, succinate, and propionate [60].

Some bacterial sequences enriched >1% were unique to either marine or terrestrial PBBRs at OLR 10 (Additional file 1: Table S3). In marine PBBRs, 4/7 were unclassified species whose genera shared critical features relative to molasses fermentation, such as strict anaerobiosis, wide capacity to ferment carbohydrates, with butyrate and/or acetate as major end products [61–64]. The most enriched of these representatives unique to marine PBBRs was an unclassified *Garciella* (OTU0010) (Additional file 1: Table S3), whose first member was isolated from seawater samples [61]. Identified bacterial species were *Lactobacillus plantarum* OTU0023 (isolated from molasses, [65]), *Desulfonispora thiosulfatigenes* (OTU0012), and *Proteiniphilum acetatigenes* (OTU0021), which, however, are not known as primary sugar-degraders [66, 67]. Sequences unique to terrestrial PBBRs were all genera common to anaerobic fermentative environments (Additional file 1: Table S3), with *L. manihotivorans* (OTU0011) first isolated from cassava sour starch fermentation [68]. The high abundance of protein or amino acid fermenters (e.g., *Aminobacterium*, *Tepidimicrobium*, *P. acetatigenes*) common or unique to either PBBRs together with carbohydrate fermenters (*Lactobacillus*, *Clostridium*) supports the hypothesis that biomass turnover represents a key functionality in sugar fermentation in brine systems.

## Conclusions

Molasses potential as renewable feedstock for bioproduction relies in its high sugar content. The inherent salinity and viscosity of this substrate represent a biotechnological barrier preventing high bioconversion rates, demanding improved bioprocessing procedures that cope with microbial biomass stability and biocatalytic activity. The present study provides critical insights into the microbial fermentation kinetics, bioprocessing, and molecular biology of brine environments which are relevant to moving towards industrial application, namely, (1) microbial communities can attain high molasses bioconversion yields into VFAs up to conductivities of 90 mS cm^−1^ irrespective of their native acclimation to salts; (2) PBBRs are ideal systems to cope with high salinities for both biomass retention and VFA production as compared to UASB reactors; (3) reduction of COD removal aimed at enhancing VFA production may be attained by adjusting salinity levels and HRT; (4) biomass turnover, rather than methanogenesis, impacts VFA production yields in saline systems; and (5) culture conditions are selected for equivalent microbial functionalities rather than community structures.

## Additional files

**Additional file 1.** Supplementary information in form of supplementary figures, tables containing enriched species per conditions and extended information on molecular analysis methodology.

**Additional file 2.** List of all bacterial sequences (Operational Taxonomic Unit, OTU) found in all samples of the study sequenced for 16S rRNA.

## Abbreviations

PBBR: packed-bed biofilm reactor
VFA: volatile fatty acid
OLR: organic loading rate
HRT: hydraulic retention time
COD: chemical oxygen demand
NMDS: non-metric multidimensional scaling
VS: volatile solids
VSS: volatile suspended solids
TS: total solids
TKN: total Kjeldahl nitrogen
DA: degree of acidification
Y_VFA_: VFA bioconversion yield
pH_i_: initial pH
$\Delta G^{01}_{{35^\circ {\text{C}}}}$: standard gibbs energy change (compensated by temperature and corrected for biological standard state)
UASB: upflow anaerobic sludge blanket
