# Supplementary material for: Efficient molasses fermentation under high salinity by inocula of marine and terrestrial origin
Source: Biotechnol Biofuels. 2017 Jan 31;10:23. doi: 10.1186/s13068-017-0701-8 (PMC5282813; doi:10.1186/s13068-017-0701-8)
Supplement: Supplementary file 1 — Additional file 1. Supplementary information in form of supplementary figures, tables containing enriched species per conditions and extended information on molecular analysis methodology. [file 13068_2017_701_MOESM1_ESM.docx]

**Efficient molasses fermentation under high salinity by inocula of marine and terrestrial origin**

Alberto Scoma^1^, Marta Coma^1, 2^, Frederiek-Maarten Kerckhof^1^, Nico Boon^1*^ and Korneel Rabaey^1*^

^1^ Center for Microbial Ecology and Technology (CMET), University of Gent, Coupure Links 653, B 9000 Gent, Belgium

^2^ Centre for Sustainable Chemical Technologies (CSCT), University of Bath, Claverton Down, BA2 7AY, Bath, United Kingdom

**Number of pages of Supporting Information:** 19

**Number of Supporting Tables:** 3

**Number of Supporting Figures:** 12

**Contents**

Figure S1. Graphical distribution of organic and inorganic fraction in raw molasses 3

Figure S2. Schematic representation of the Packed Bed Biofilm Reactor set up 4

Figure S3. CO_2_ gas accumulation during the enrichment of terrestrial and marine cultures using molasses. 5

Figure S4. H_2_ to CO_2_ ratio in terrestrial and marine cultures operated at ORL 5 – pHi 7.........6

Figure S5. Fermentation kinetics of acetic (C2), propionic (C3), isobutyric (iC4) and butyric (C4) acid in terrestrial and marine cultures during enrichments on molasses............................7

Figure S6. Kinetics of total VFAs accumulation in terrestrial and marine cultures during the enrichment.. 8

Figure S7. pH patterns in terrestrial and marine cultures during the enrichment. 9

Figure S8. Volatile solids content in terrestrial and marine cultures during the enrichment 10

Figure S9. Box and whiskerplots of the Inverse Simpson (1/D) diversity index dependent upon OLR and inoculum source...............................................................................................11

Figure S10. Box and whiskerplots of the Inverse Simpson (1/D) diversity index depending upon the OLR...........................................................................................................................12

Figure S11. Collector curve for the inverse Simpson (1/D) index as calculated by mothur 1.35.1 on the dataset with singleton OTUs included................................................................13

Table S1. OTUs enriched >1% which were shared by marine and terrestrial PBBRs at the end of the continuous mode of operation when reactors were fed with 10 g_COD_ L^-1^ d^-1^ (OLR 10), in both biolfilm and effluents (bulk liquid phase). 14

Table S3. OTUs enriched >1% which were unique to either marine or terrestrial PBBRs at the end of the continuous mode of operation when reactors were fed with 10 g_COD_ L^-1^ d^-1^ (OLR 10), in both biolfilm and effluents (bulk liquid phase). 15

Supplementary Note S1. Molecular analysis and bioinformatics 16

Figure S12. Rarefraction curves generated using vegan on the OTU table after removal of OTUs with only a single sequence read in one of the sample (i.e. “singleton” OTUs)...........18

Supplementary References 19

# Figure S1. Graphical distribution of organic and inorganic fraction in raw molasses

Organic (left) and inorganic (right) distribution of raw molasses composition. Glucose was the only sugar measured, thus other carbohydrates might be included in the soluble COD [Other sCOD].


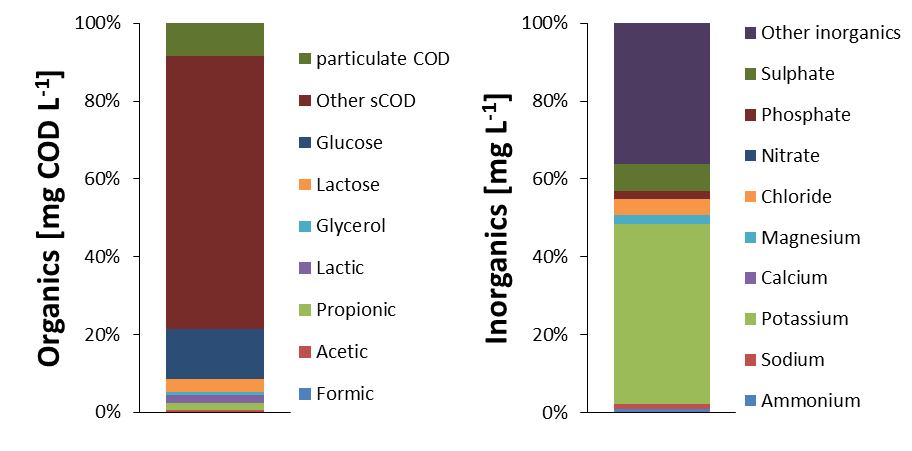


# Figure S2. Schematic representation of the Packed Bed Biofilm Reactor set up

Packed Bed Biofilm Reactor (PBBR, middle) with diagrams of influent, effluent, recirculation and biogas flows. Influent was continuously stirred and kept at 4 °C. Effluent was withdrawn by overflow. pH was monitored and controlled within the recirculation line, which followed a top to bottom flow. Biogas was collected from the top of the reactor in an acidified water column to avoid CO_2_ absorption.


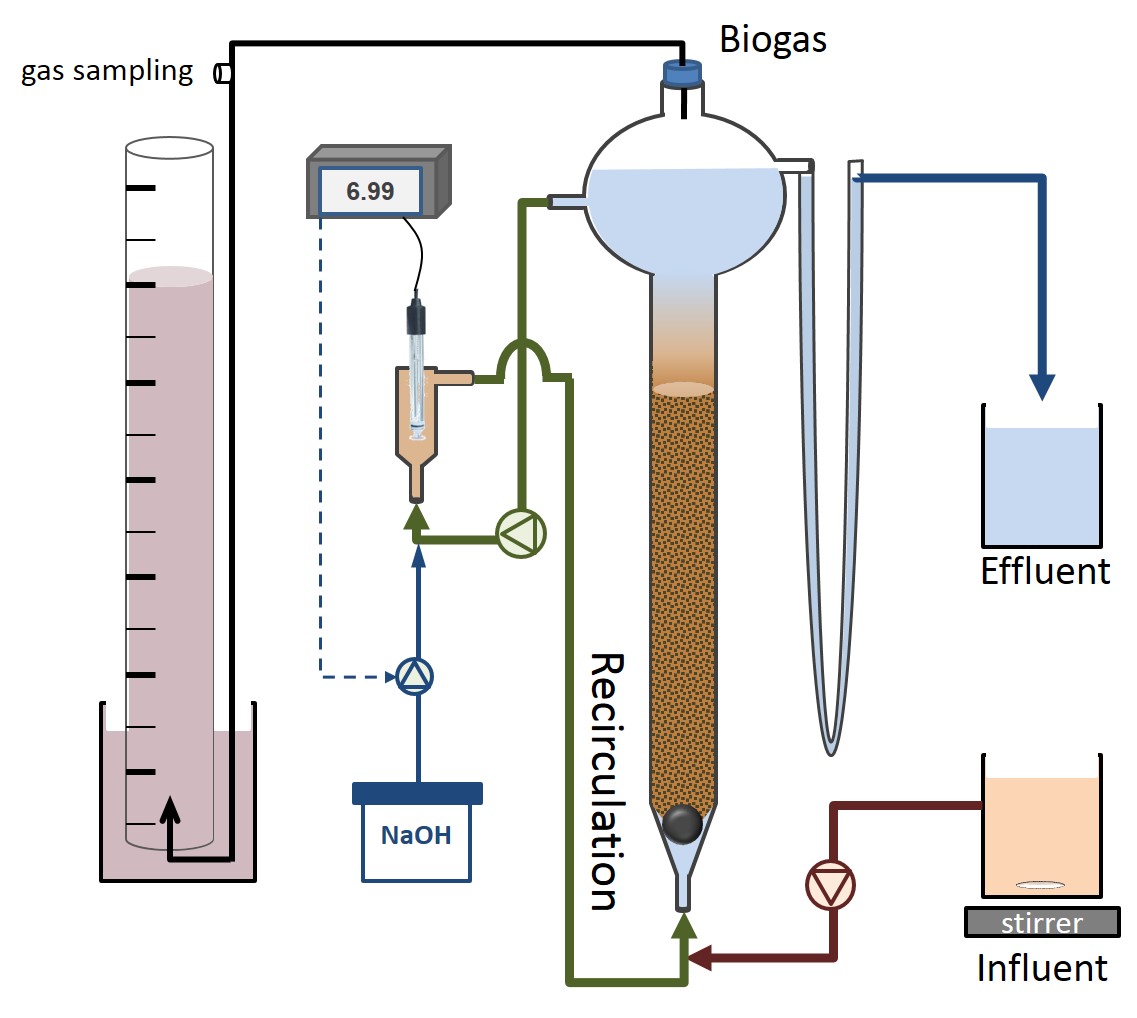


# Figure S3. CO_2_ gas accumulation during the enrichment of terrestrial and marine cultures using molasses.

Cultures were tested in batch and had different initial pH (pH_i_) (either 6 or 7) and organic loading rates (OLR) (either 1 or 5 g_COD_ L^-1^ d^-1^, equivalent to an initial content of 7 or 35 g_COD_ L^-1^, respectively). Temperature was set to 35 °C. Cultures were tested for 7 days, after which 10% liquid volume was withdrawn and incubated again with fresh medium for another 7 days. Hence, the enrichment consisted of 3 consecutive batches of 1 week each. Marine cultures were provided with 23 g L^-1^ NaCl to maintain their original salinity at all conditions. Error bars represent standard deviations of 3 independent biological replicates. Keys reported in the graph.


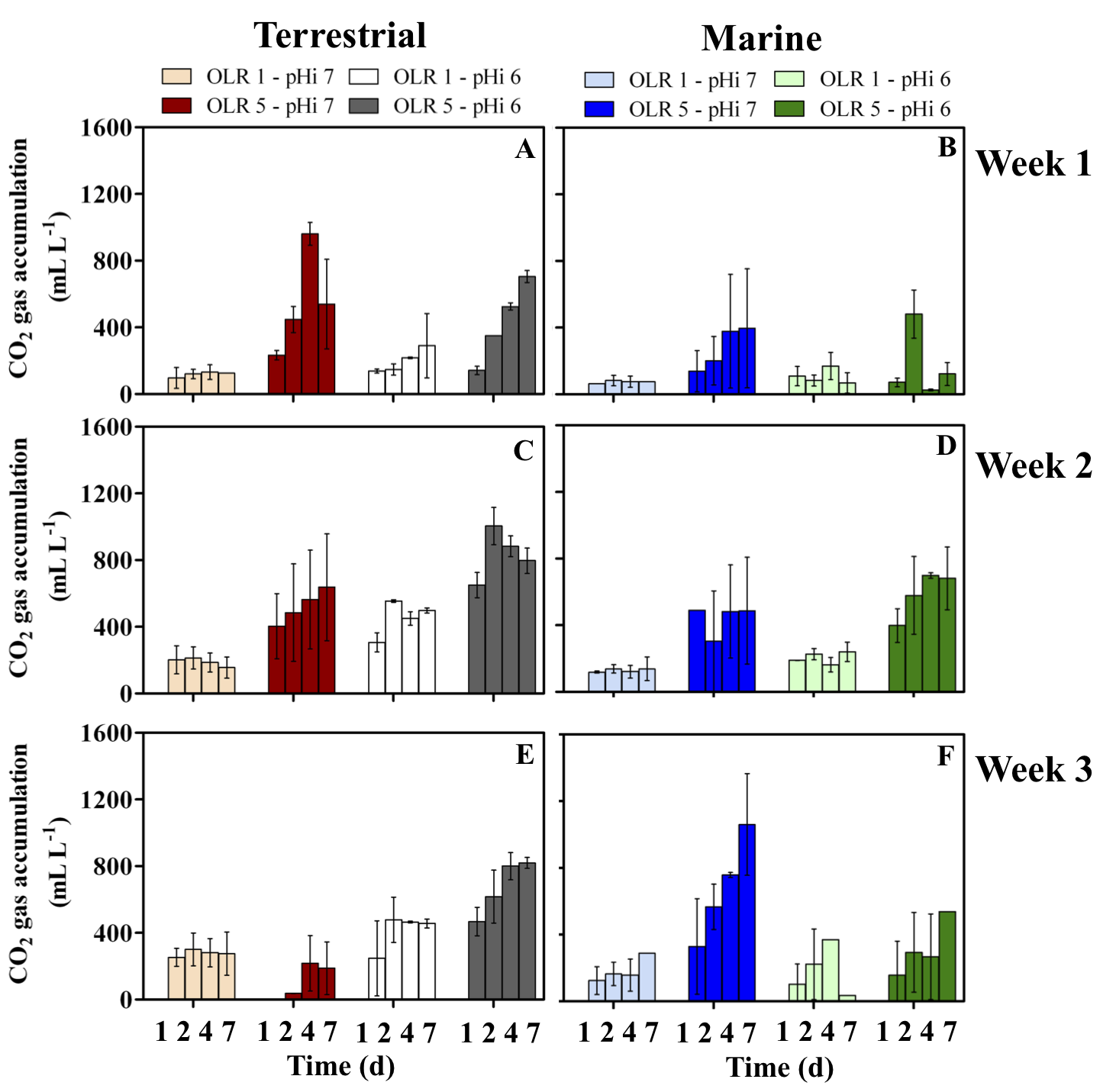


# Figure S4. H_2_ to CO_2_ ratio in terrestrial and marine cultures operated at ORL 5 – pHi 7.

Week 1, 2 and 3 indicate the sequential fermentation batches that constituted the enrichment. Results are the average of experiments done in three independent replicates.


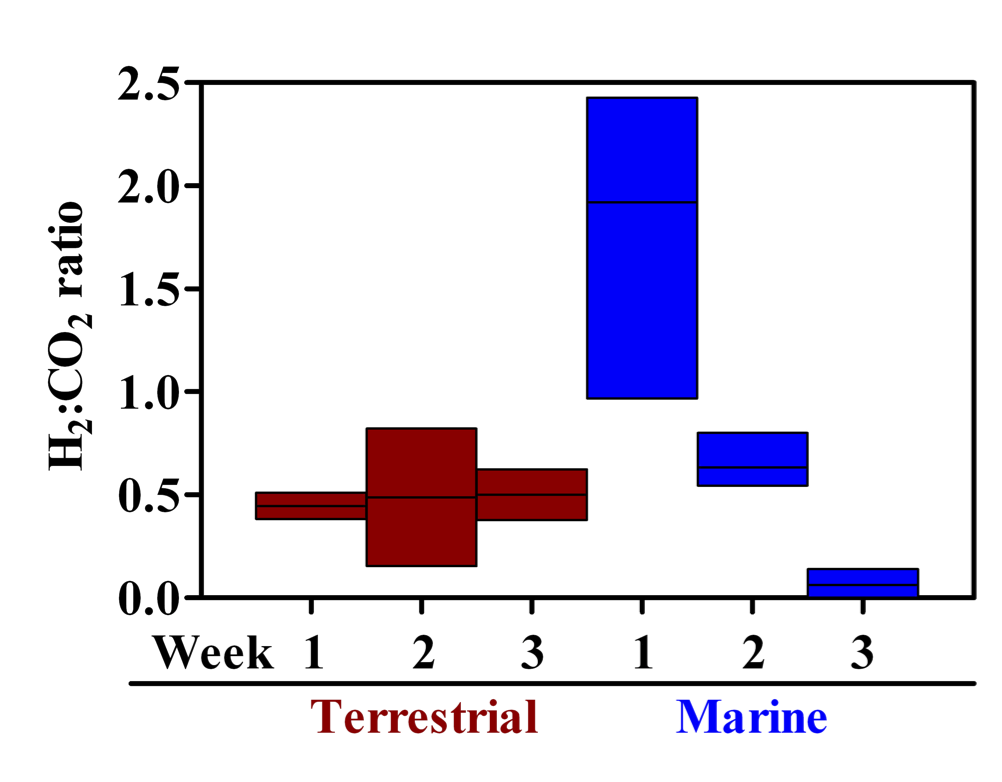


# Figure S5. Fermentation kinetics of acetic (C2), propionic (C3), isobutyric (iC4) and butyric (C4) acid in terrestrial and marine cultures during enrichments on molasses.

Cultures were tested in batch and had different initial pH (pH_i_) (either 6 or 7) and organic loading rates (OLRs) (either 1 or 5 g_COD_ L^-1^ d^-1^, equivalent to an initial content of 7 or 35 g_COD_ L^-1^, respectively). Temperature was set to 35 °C. Cultures were tested for 7 days, after which 10% liquid volume was withdrawn and incubated again with fresh medium for another 7 days. Hence, the enrichment consisted of 3 consecutive batches of 1 week each. Marine cultures were provided with 23 g L^-1^ NaCl to maintain their original salinity at all conditions. Error bars represent standard deviations of 3 independent biological replicates. Keys reported in the graph. Sampling days for each VFA were 1, 2, 4 and 7 days, as reported in the X axis.


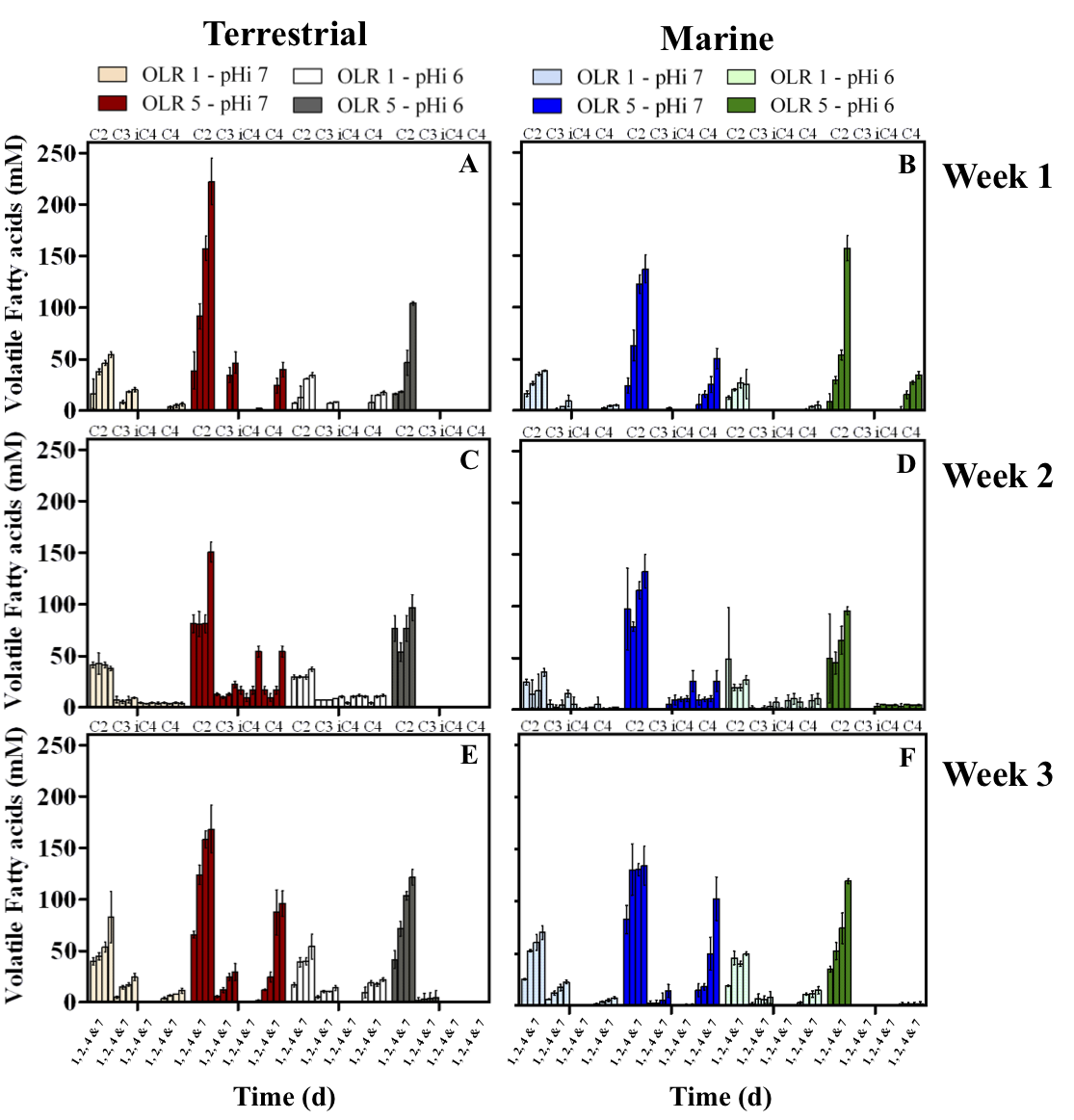


# Figure S6. Kinetics of total VFAs accumulation in terrestrial and marine cultures during the enrichment. Week 1, 2 and 3 indicate the sequential fermentation batches that constituted the enrichment. Mean values are the average of experiments done in three independent replicates. Error bars indicate standard deviation from the mean. Keys reported in the graph.


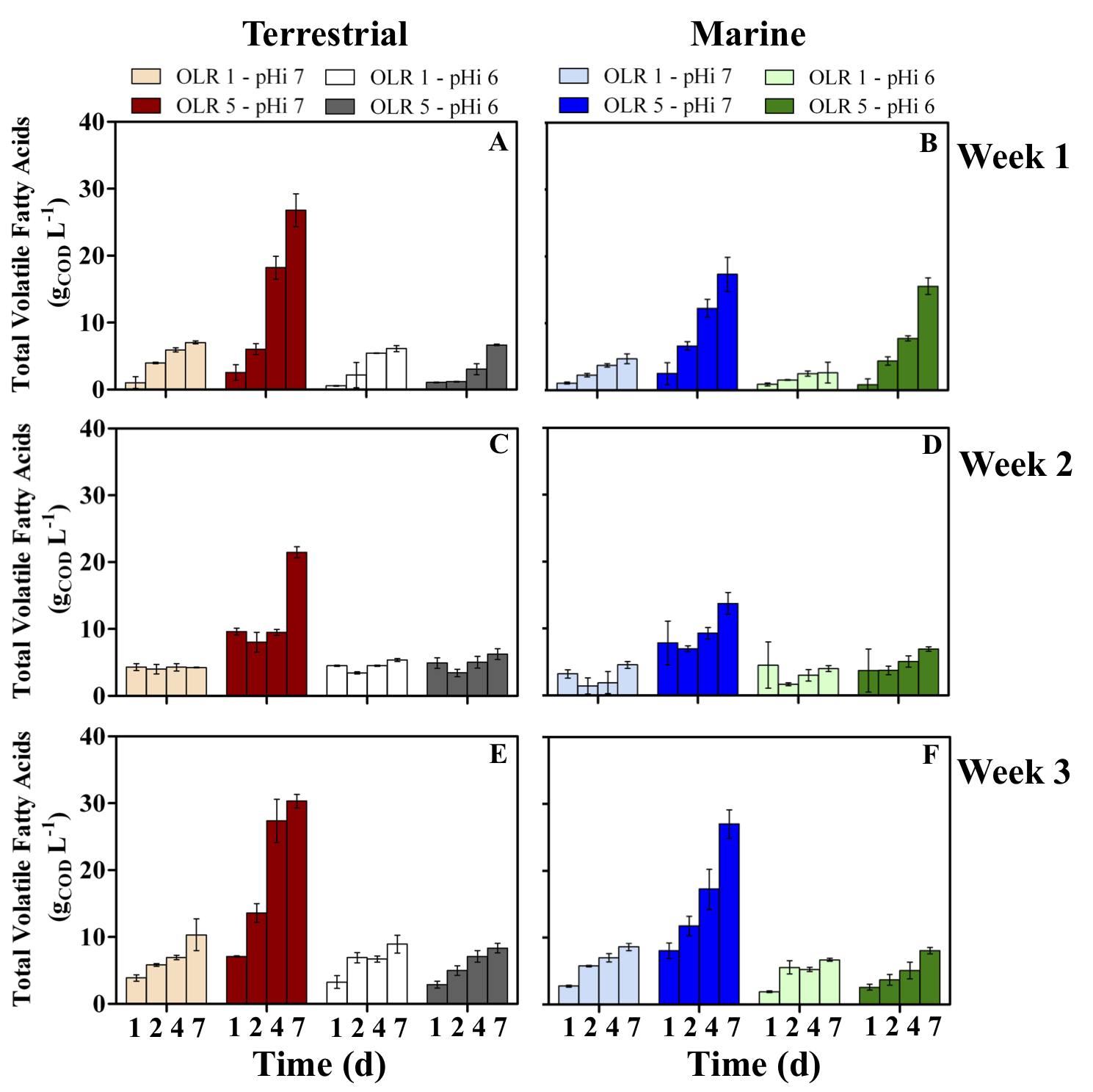


# Figure S7. pH patterns in terrestrial and marine cultures during the enrichment.

Initial pH was set to either 6 or 7 (dotted line) and manually corrected to such values at any sampling point. Week 1, 2 and 3 indicate the sequential fermentation batches that constituted the enrichment. Mean values are the average of experiments done in three independent replicates. Error bars indicate standard deviation from the mean. Keys reported in the graph.


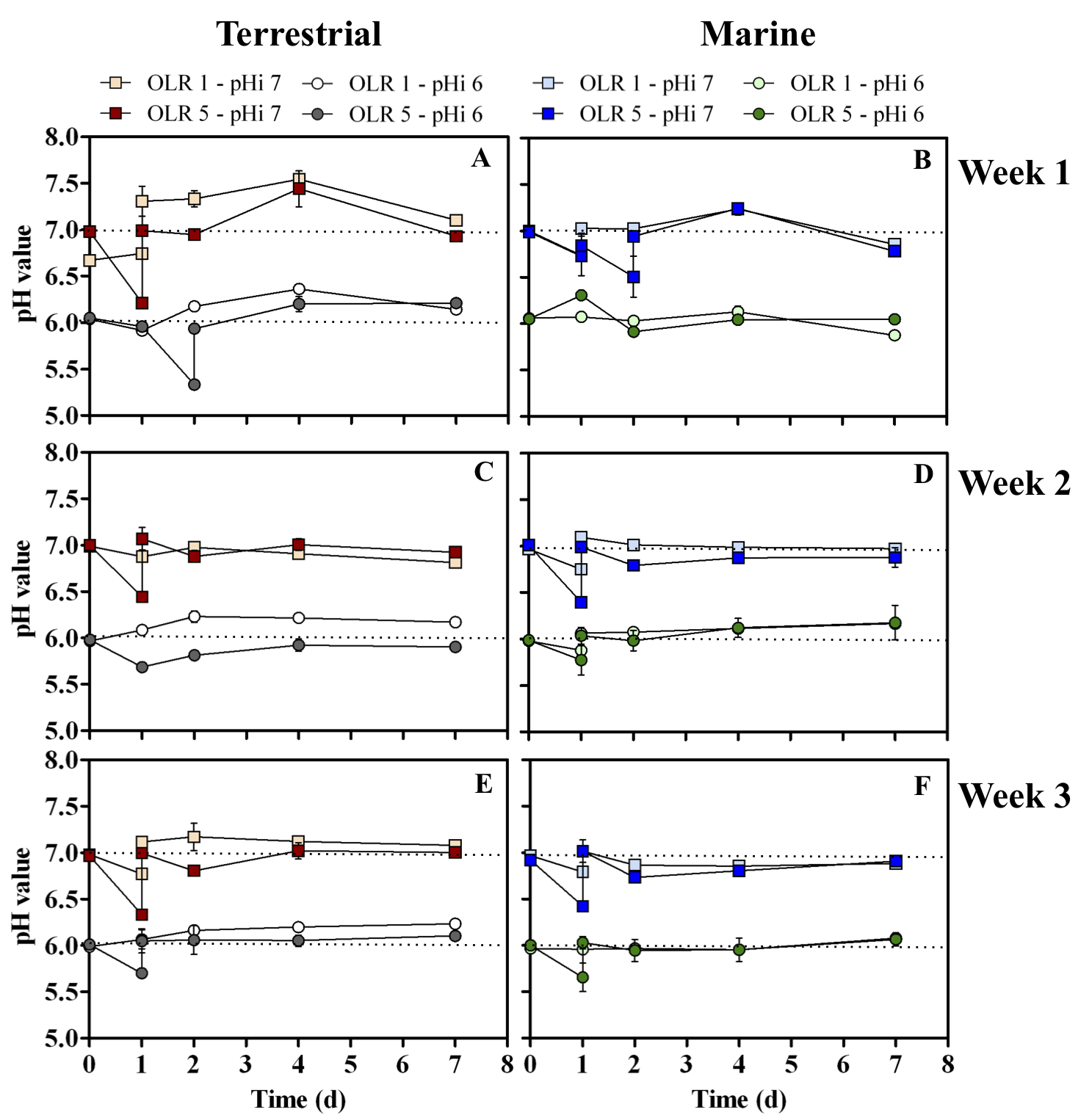


# Figure S8. Volatile suspended solids (VSS) content in terrestrial and marine cultures during the enrichment. Week 1, 2 and 3 indicate the sequential fermentation batches that constituted the enrichment. Mean values are the average of experiments done in three independent replicates. Error bars indicate standard deviation from the mean. Keys reported in the graph.

**
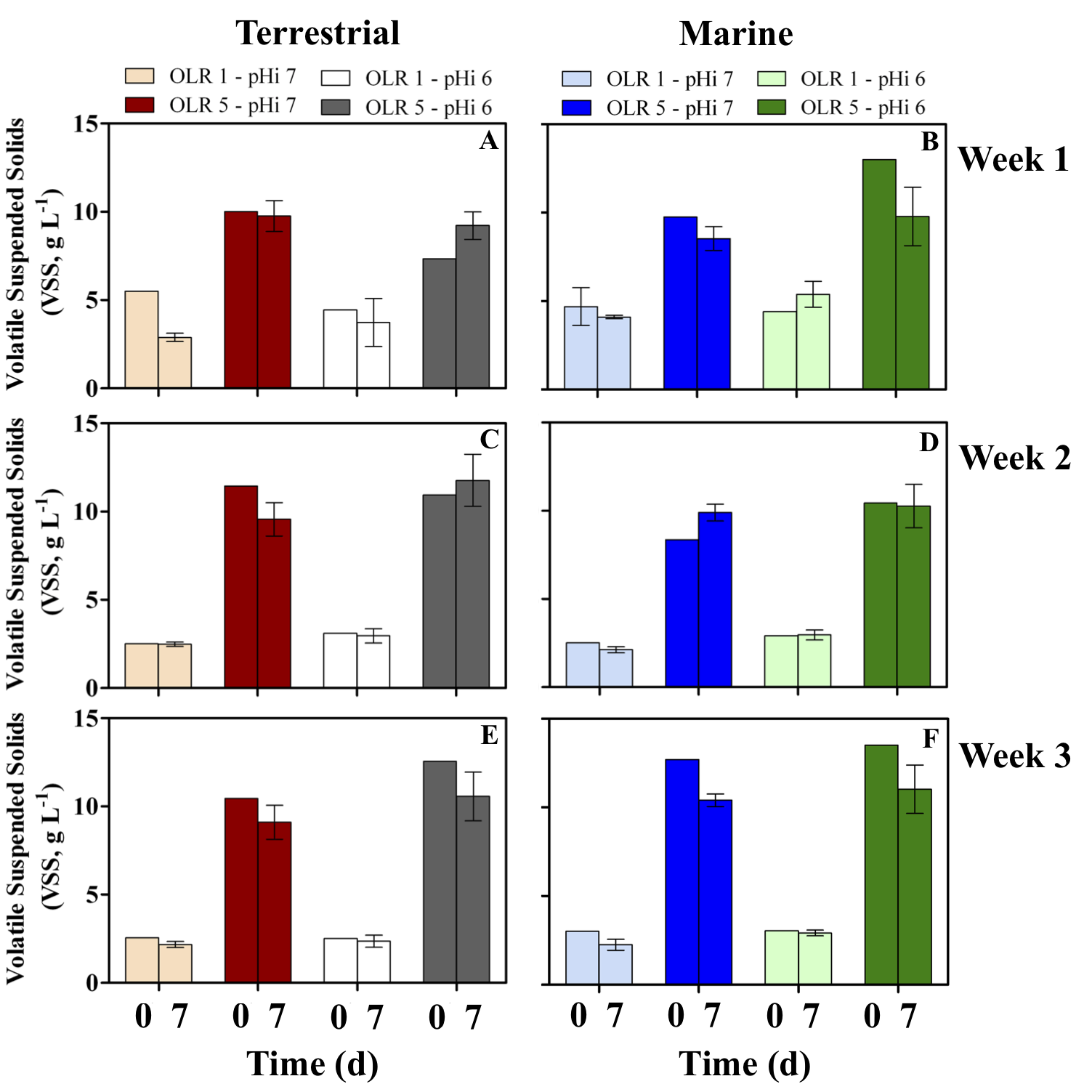
**

**Figure S9. Box and whiskerplots of the Inverse Simpson (1/D) diversity index dependent upon OLR and inoculum source.**
The analysis was based on effluent samples collected from PBBRs at the end of the operation.


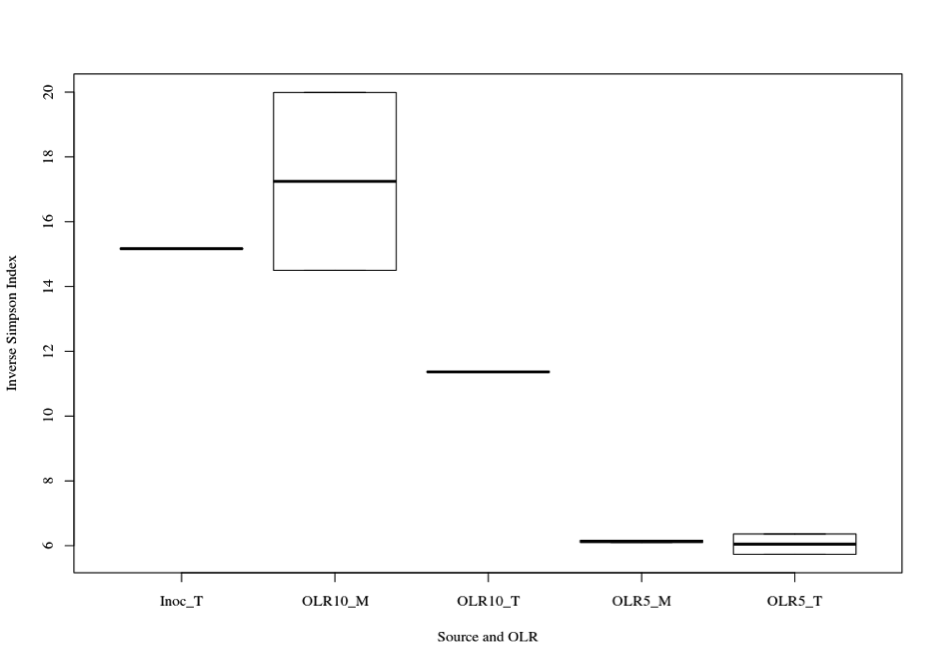


**Figure S10. Box and whiskerplots of the Inverse Simpson (1/D) diversity index depending upon the OLR**.
The analysis was based on effluent samples collected from PBBRs at the end of the operation.


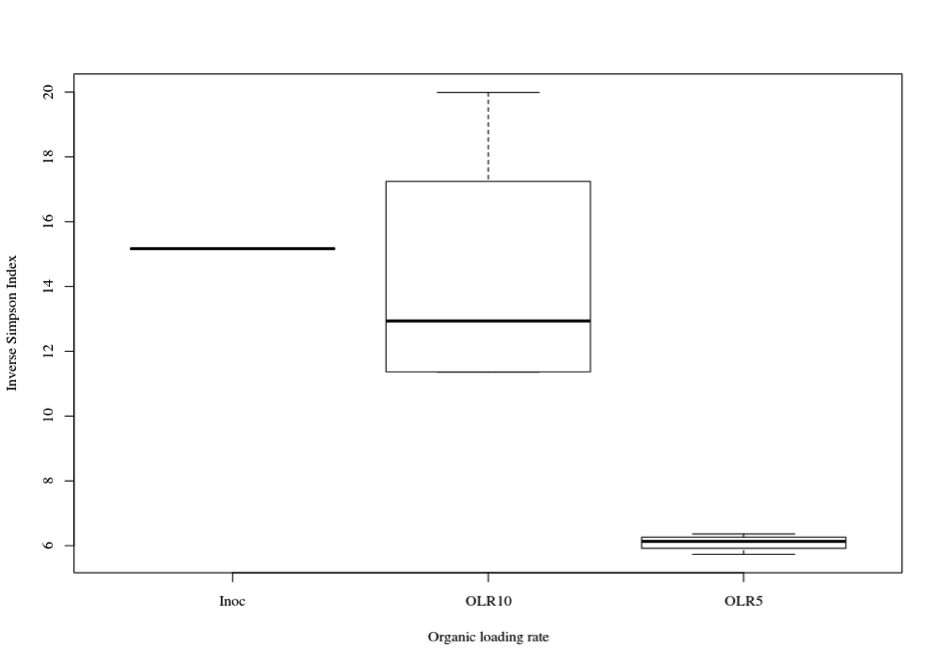


**Figure S11. Collector curve for the inverse Simpson (1/D) index as calculated by mothur 1.35.1 on the dataset with singleton OTUs included**. Error bars indicate 95% confidence intervals on the point estimates of the index.

# Table S1. OTUs enriched >1% which were shared by marine and terrestrial PBBRs at the end of the continuous mode of operation when reactors were fed with 10 g_COD_ L^-1^ d^-1^ (OLR 10), in both biolfilm and effluents (bulk liquid phase).

Values represent the relative abundance (%) with respect to the total number of sequences of each sample. Numbers in brackets represent the sequence identity (%) with respect to 16S ribosomal sequences withdrawn from Silva database (see Note S1).

| **Marine** | **Terrestrial** | **OTU** | **Taxonomy** | | **Shared by bulk and biofilm?** |
| --- | --- | --- | --- | --- | --- |
| **OLR 10 biofilm** | |  | **Genus** | **species** |  |
| 6.05 | 20.64 | Otu0003 | Bacteroides(100) | coprosuis(100) | Yes |
| 9.33 | 16.06 | Otu0005 | Aminobacterium(100) | unclassified(100) | Yes |
| 10.81 | 8.26 | Otu0004 | Bacteroides(100) | coprosuis(100) | Yes |
| 2.32 | 4.84 | Otu0002 | Bacteroides(100) | coprosuis(100) | Yes |
| 2.42 | 3.99 | Otu0009 | Tissierella_Soehngenia(98) | unclassified(98) | Yes |
| 9.18 | 2.90 | Otu0007 | Tepidimicrobium(100) | unclassified(100) | Yes |
| 5.51 | 2.90 | Otu0011 | Lactobacillus(100) | manihotivorans(100) | No |
| 2.32 | 1.88 | Otu0025 | Peptoniphilus(100) | unclassified(100) | No |
| 3.22 | 1.60 | Otu0023 | Lactobacillus(100) | plantarum(100) | No |
| **Marine** | **Terrestrial** | **OTU** | **Taxonomy** | | **Shared by bulk and biofilm?** |
| **OLR 10 end (bulk)** | |  | ***Genus*** | ***species*** |  |
| 9.30 | 19.44 | Otu0004 | Bacteroides(100) | coprosuis(100) | Yes |
| 9.51 | 14.02 | Otu0017 | Bacillus(100) | oleronius(98) | No |
| 1.61 | 12.58 | Otu0003 | Bacteroides(100) | coprosuis(100) | Yes |
| 7.73 | 7.01 | Otu0005 | Aminobacterium(100) | unclassified(100) | Yes |
| 3.17 | 4.37 | Otu0007 | Tepidimicrobium(100) | unclassified(100) | Yes |
| 2.04 | 3.88 | Otu0002 | Bacteroides(100) | coprosuis(100) | Yes |
| 1.13 | 2.71 | Otu0009 | Tissierella_Soehngenia(98) | unclassified(98) | Yes |
| 2.22 | 2.03 | Otu0021 | Proteiniphilum(100) | acetatigenes(100) | No |
| 4.39 | 1.21 | Otu0012 | Desulfonispora(100) | thiosulfatigenes(100) | No |
| 1.43 | 1.13 | Otu0019 | Bacteroides(100) | unclassified(97) | No |

# Table S3. OTUs enriched >1% which were unique to either marine or terrestrial PBBRs at the end of the continuous mode of operation when reactors were fed with 10 g_COD_ L^-1^ d^-1^ (OLR 10), in both biolfilm and effluents (bulk liquid phase).

Values represent the relative abundance (%) with respect to the total number of sequences of each sample. Numbers in brackets represent the sequence identity (%) with respect to 16S ribosomal sequences withdrawn from Silva database (see Note S1).

| **Marine** | | **OTU** | **Taxonomy** | |
| --- | --- | --- | --- | --- |
| **OLR 10 end (bulk)** | **OLR 10 biofilm** |  | ***Genus*** | ***species*** |
| 14.25 | 8.77 | Otu0010 | Garciella(100) | unclassified(100) |
| 2.78 | 3.22 | Otu0023 | Lactobacillus(100) | plantarum(100) |
| 1.22 | 3.12 | Otu0013 | Anaerococcus(100) | unclassified(100) |
| 4.39 | 2.53 | Otu0012 | Desulfonispora(100) | thiosulfatigenes(100) |
| 2.22 | 2.36 | Otu0021 | Proteiniphilum(100) | acetatigenes(100) |
| 1.30 | 1.53 | Otu0041 | Coprococcus(100) | unclassified(100) |
| 1.43 | 1.14 | Otu0019 | Bacteroides(100) | unclassified(97) |
| **Terrestrial** | | **OTU** | **Taxonomy** | |
| **OLR 10 end (bulk)** | **OLR 10 biofilm** |  | ***Genus*** | ***species*** |
| 5.46 | 5.31 | Otu0016 | Clostridium(100) | unclassified(100) |
| 1.43 | 2.62 | Otu0027 | Tepidimicrobium(100) | unclassified(100) |
| 1.21 | 2.90 | Otu0011 | Lactobacillus(100) | manihotivorans(100) |

# Supplementary Note S1. Molecular analysis and bioinformatics

*Amplification of DNA samples*

In a first step, the 16S rRNA gene V3-V4 hypervariable regions were amplified by PCR using primers derived from [1], with a slight modification to the reverse primer by introducing another wobble position (K) to make it more universal. The PCR mix included 1 ng DNA extract, 15 pmol of both the forward primer 341F 5'- NNNNNNNNNTCCTACGGGNGGCWGCAG and reverse primer 785R 5'- NNNNNNNNNNTGACTACHVGGGTATCTAAKCC in 20 µL volume of MyTaq buffer containing 1.5 units MyTaq DNA polymerase (Bioline) and 2 µl of BioStabII PCR Enhancer (Sigma). For each sample, the forward and reverse primers had the same unique 10-nt barcode sequence (represented by (N)10 in the primer sequences). PCRs were carried out for 20 cycles as follows: 2 min 96 °C pre-denaturation; 96 °C for 15 s, 50 °C for 30 s, 70 °C for 90 s. If needed PCRs showing low yields were further amplified for 5 cycles. DNA concentration of amplicons of interest was determined by gel-electrophoresis. About 20 ng amplicon DNA of each sample were pooled for up to 48 samples carrying different barcodes. The amplicon pools were purified with one volume AMPure XP beads (Agencourt) to remove primer dimer and other small mispriming products, followed by additional purification on MinElute columns (Qiagen). Finally, about 100 ng of each purified amplicon pool DNA was used to construct Illumina libraries by means of adaptor ligation using the Ovation Rapid DR Multiplex System 1-96 (NuGEN). Illumina libraries were pooled and size-selected by preparative gel-electrophoresis. Sequencing was performed on an Illumina MiSeq using version 3 chemistry (Illumina).

*Bioinformatics for Illumina sequences*

The mothur software package (v.1.35.1) and guidelines developed by Patrick Schloss (<http://www.mothur.org/wiki/MiSeq_SOP>, last accessed April 2016) were used to process the obtained amplicon sequencing data [2]. First, forward and reverse reads were assembled into contigs by means of a heuristic approach taking the Phred quality scores into account (150796 contigs). Contigs with ambiguous base calls or unsatisfying overlap were removed, retaining 55.2% of the data. The remaining sequences, with a length between 408-482 bases, were aligned to mothur formatted silva .seed release 119 alignment database trimmed between positions 6388-25316, to be compatible with the 341F-785R primers. Any sequences not aligning within this region or containing homopolymer stretches of length more than 12 were removed, resulting in additional 12.2% of the data being culled out. Data was then pre-clustered allowing up to 4 differences between sequences. Chimera check was performed with UCHIME, to finally retain only 38.15% of the original reads. Sequences were classified by means of a naïve Bayesian classifier, against the RDP 16S rRNA gene training set, version 14, 80% cut-off for the pseudobootstrap confidence score. Taxa with annotation Chloroplast, Mitochondria, unknown, *Archaea* or *Eukaryota* at the kingdom level were excluded, however no sequences were classified as such. A pairwise distance matrix was calculated between all remaining reads (with a 35% dissimilarity cut-off) after which average linkage clustering into OTU's at the 3% dissimilarity level was executed. Accordingly, an OTU was defined in this manuscript as a collection of sequences with a length between 410-440 nt that are found to be more than 97% similar to one another in the V3-V4 hypervariable region of their 16S rRNA gene after applying hierarchical clustering [3–5]. All OTUs with only one read in one sample (singletons) were removed. Finally, 215 OTUs were retained among all samples. OTUs were classified based upon the consensus of their individual sequence classifications by means of the SILVA non-redundant database (release 119). Collector curves were generated to assess the dependency of alpha-diversity indices to sampling depth. Only indices that were stable over the entire range of our sample sizes were incorporated (Inverse Simpson diversity index, Shannon entropy diversity index, Shannon eveness index and Simpson eveness index). Rarefaction curves (Fig. S12) show that for most samples an adequate depth of sampling was obtained. Al beta-diversity analyses were performed in R (version 3.2.4 Revised (2016-03-16 r70336), [http://www.r-project.org](http://www.r-project.org/)) using the vegan package (version 2.3-5, [6]).

**Figure S12. Rarefraction curves generated using vegan on the OTU table after removal of OTUs with only a single sequence read in one of the sample (i.e. “singleton” OTUs).** The label coding indicates terrestrial (T) or marine (M) inoculum, organic loading rate (OLR) 5 or 10 and at which point the sampling was performed (start/end/bulk/biofilm).

# Supplementary References

1. Klindworth A, Pruesse E, Schweer T, Peplies J, Quast C, Horn M, et al. Evaluation of general 16S ribosomal RNA gene PCR primers for classical and next-generation sequencing-based diversity studies. Nucleic Acids Res. 2013;41.

2. Kozich JJ, Westcott SL, Baxter NT, Highlander SK, Schloss PD. Development of a dual-index sequencing strategy and curation pipeline for analyzing amplicon sequence data on the miseq illumina sequencing platform. Appl. Environ. Microbiol. 2013;79:5112–20.

3. Chen W, Zhang CK, Cheng Y, Zhang S, Zhao H. A Comparison of Methods for Clustering 16S rRNA Sequences into OTUs. PLoS One. 2013;8.

4. Wang X, Cai Y, Sun Y, Knight R, Mai V. Secondary structure information does not improve OTU assignment for partial 16s rRNA sequences. ISME J. [Internet]. 2012;6:1277–80. Available from: http://www.nature.com/doifinder/10.1038/ismej.2011.187

5. Schloss PD, Westcott SL. Assessing and improving methods used in operational taxonomic unit-based approaches for 16S rRNA gene sequence analysis. Appl. Environ. Microbiol. 2011;77:3219–26.

6. Oksanen J, Blanchet F, Kindt R, Legendre P, O’Hara R. Vegan: community ecology package. R Packag. 2.3-3 [Internet]. 2016;Available at: https://cran.r – project.org/web/packa. Available from: http://cran.r-project.org/package=vegan
